# Supplementary material for: Genome-wide aggregated trans-effects on risk of type 1 diabetes: A test of the “omnigenic” sparse effector hypothesis of complex trait genetics
Source: Am J Hum Genet. 2023 May 9;110(6):913–26. doi: 10.1016/j.ajhg.2023.04.003 (PMC10257008; doi:10.1016/j.ajhg.2023.04.003)
Supplement: Document S1. Figure S1 and Tables S1, S4, and S6 [file mmc1.pdf]

The American Journal of Human Genetics, Volume *110*

Supplemental information

Genome-wide aggregated *trans*-effects on risk of type 1 diabetes: A test of the “omnigenic” sparse effector hypothesis of complex trait genetics

Andrii Iakovliev, Stuart J. McGurnaghan, Caroline Hayward, Marco Colombo, Debby Lipschutz, Athina Spiliopoulou, Helen M. Colhoun, and Paul M. McKeigue

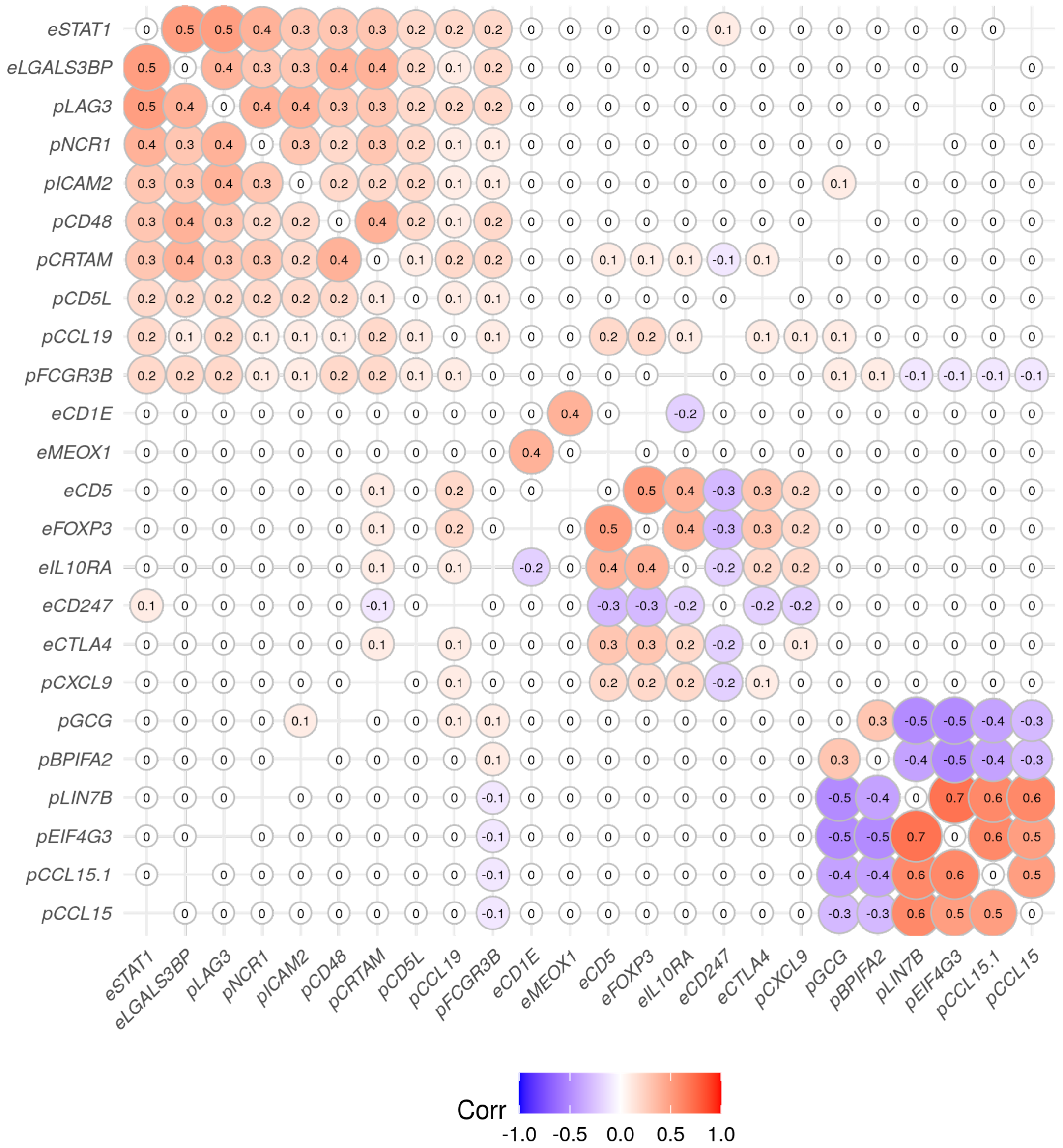

Fig S1. Heat map of correlations (in control group) between *trans*- scores for putative effector genes

The correlations between eQTL scores for genes identified in Table 2 and pQTL scores for genes identified in Table 4 is reported. Rows and columns are ordered by hierarchical clustering. The prefixes e and p denote scores for gene expression scores and circulating protein levels respectively.

Table S1. *cis-* eQTL or pQTLassociations with T1D detected at $p < 0.01$ in the 12q13 and 12q24 regions.

Gene	Start position (Mb)	*cis*-eQTL		*cis*-pQTL	
		Log odds ratio	p-value	Log odds ratio	p-value
**12q13 region**					
RAB5B	55.97	0.14	9×10^{-14}	.	.
ERBB3	56.08	.	.	-0.14	6×10^{-13}
ERBB3	56.08	.	.	-0.13	3×10^{-11}
IL23A	56.33	-0.16	2×10^{-17}	.	.
GLS2	56.47	-0.05	0.006	.	.
**12q24 region**					
ISCU	108.56	-0.01	0.4	0.14	1×10^{-12}
SELPLG	108.62	0.02	0.3	0.11	2×10^{-9}
GLTP	109.85	.	.	0.11	6×10^{-9}
PPTC7	110.53	-0.14	4×10^{-13}	.	.
PHETA1	111.36	-0.06	0.002	.	.
SH2B3	111.41	0.08	2×10^{-5}	.	.
ALDH2	111.77	0.01	0.5	0.10	1×10^{-7}
TRAFD1	112.13	0.11	4×10^{-8}	.	.
OAS3	112.94	-0.05	0.005	.	.
OAS2	112.98	-0.06	6×10^{-4}	.	.
SDSL	113.42	0.11	2×10^{-9}	0.03	0.2
TBX5	114.35	.	.	0.12	1×10^{-9}

Table S4. Associations of T1D with genome-wide scores for regulatory T cell phenotypes computed using weights from SardiNIA study[1].

	Absolute count		% of parent lineage		% of grandparent lineage	
Cell phenotype	Log odds ratio	p-value	Log odds ratio	p-value	Log odds ratio	p-value
CD39+ (resting & activated) CD4+ Treg	.	.	-0.03	0.08	-0.02	0.2
CD39+ (secreting & activated) CD4+ Treg	-0.02	0.2	-0.01	0.3	-0.01	0.4
CD39+ CD4+ Treg	-0.01	0.7	-0.01	0.4	0.00	0.8
CD39+ activated CD4+ Treg	-0.03	0.07	-0.01	0.3	-0.02	0.3
CD39+ resting CD4+ Treg	-0.02	0.1	-0.03	0.02	-0.01	0.4
CD39+ secreting CD4+ Treg	-0.02	0.2	-0.02	0.2	0.00	0.9
CD4+ Treg	-0.08	0.03	-0.06	0.03	-0.01	0.8
activated CD4+ Treg	.	.	-0.05	0.3	-0.01	0.7
resting & activated CD4+ Treg	0.03	0.3	0.02	0.3	0.00	0.9
resting CD4+ Treg	0.02	0.6	-0.01	0.7	0.02	0.6
secreting & activated CD4+ Treg	-0.07	0.008	0.00	0.8	-0.05	0.04
secreting CD4+ Treg	-0.05	0.08	-0.01	0.6	-0.02	0.4

Immune cell phenotypes are defined in Orrù et al. (2013) supplementary information[1].

Table S6. *Trans*-pQTLs near T1D-associated genes contributing to *trans*- scores in Table 4.

Chr	Start position (Mb)	End position (Mb)	Genes to which T1D association with SNPs within 200 kb of the *trans*-pQTL region was attributed	Genes encoding proteins with T1D-associated *trans*- scores
1	113.53	113.99	*PTPN22, MAGI3, PHTF1, RSBN1*	*CCL19, CRTAM, CXCL9*
1	206.87	206.97	*IL10*	*BPIFA2*
2	60.62	60.62	*BCL11A, KIAA1841*	*CXCL9*
2	191.09	191.14	*STAT4*	*CRTAM*
2	203.88	203.92	*CTLA4*	*CCL19*
3	45.97	47.54	*CCR9*	*CXCL9, CRTAM, CD48*
4	122.71	122.71	*KIAA1109*	*CXCL9*
5	86.75	86.79	*AC008394.1*	*CCL15*
6	35.42	35.42	*TULP1*	*CXCL9*
7	20.27	20.27	*ITGB8*	*GCG*
7	50.23	50.41	*IKZF1, FIGNL1*	*FCGR3B, LAG3, CD5L*
8	126.91	127.22	*PVT1*	*CD5L*
9	133.17	133.73	*CEL, RALGDS, OBP2B*	*ICAM2, CCL15, BPIFA2, GCG*
10	88.05	88.05	*RNLS*	*ICAM2*
11	2.51	2.53	*INS, TH*	*CXCL9*
11	128.34	128.54	*FLI1*	*CRTAM*
12	9.07	9.07	*KLRG1, PHC1*	*CD48*
12	110.82	112.88	*ATXN2, BRAP*	*CD5L, NCR1, CD48, CXCL9, LAG3, FCGR3B, CRTAM, ICAM2, CCL19*
12	112.70	112.75	*ATXN2*	*LAG3*
13	98.79	98.79	*GPR183*	*CXCL9*
14	100.70	100.72	*AL117190.2*	*NCR1*
16	30.16	31.14	*MAPK3*	*CD5L*
17	6.83	7.64	*ACAP1, DNAH2*	*CD48, FCGR3B, LAG3, CRTAM, CD5L*
17	39.24	39.97	*GSDMB*	*FCGR3B*
17	45.11	46.79	*STH, NSF*	*CCL15, CD48, CD5L, CRTAM*
17	47.28	48.25	*PRR15L, SKAP1*	*FCGR3B, GCG*
19	46.35	46.80	*PRDK2*	*NCR1, FCGR3B*
19	48.10	49.12	*FUT2*	*CCL15, BPIFA2, GCG, FCGR3B*
21	42.17	42.19	*UBASH3A*	*CXCL9*
21	42.88	42.88	*CFAP410*	*GCG*
22	29.73	30.20	*LIF*	*CCL19*

Supplementary references

1. Orrù, V., Steri, M., Sole, G., Sidore, C., Virdis, F., Dei, M., Lai, S., Zoledziewska, M., Busonero, F., Mulas, A., et al. (2013). Genetic Variants Regulating Immune Cell Levels in Health and Disease. Cell *155*, 242–256. 10.1016/j.cell.2013.08.041.
2. Roederer, M., Quaye, L., Mangino, M., Beddall, M.H., Mahnke, Y., Chattopadhyay, P., Tosi, I., Napolitano, L., Terranova Barberio, M., Menni, C., et al. (2015). The Genetic Architecture of the Human Immune System: A Bioresource for Autoimmunity and Disease Pathogenesis. Cell *161*, 387–403. 10.1016/j.cell.2015.02.046.
