# Supplementary material for: Genome-wide aggregated trans-effects on risk of type 1 diabetes: A test of the “omnigenic” sparse effector hypothesis of complex trait genetics
Source: Am J Hum Genet. 2023 May 9;110(6):913–26. doi: 10.1016/j.ajhg.2023.04.003 (PMC10257008; doi:10.1016/j.ajhg.2023.04.003)
Supplement: Document S2. Article plus supplemental information [file mmc5.pdf]

Genome-wide aggregated *trans*-effects on risk of type 1 diabetes: A test of the "omnigenic" sparse effector hypothesis of complex trait genetics

Graphical abstract

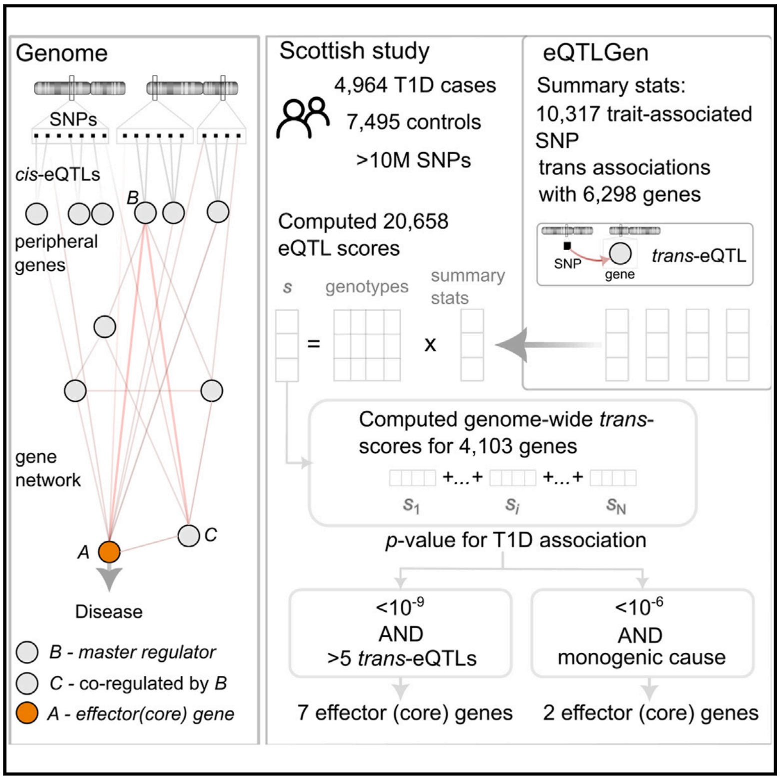

Authors

Andrii Iakovliev,
Stuart J. McGurnaghan,
Caroline Hayward, ...,
Athina Spiliopoulou,
Helen M. Colhoun, Paul M. McKeigue

Correspondence

paul.mckeigue@ed.ac.uk

Iakovliev et al. investigate whether the effects of common SNPs on risk of type 1 diabetes are mediated through *trans*-effects on expression of core genes. By testing for association with genotypic scores that aggregate predicted *trans*-effects, they identify a set of putative core genes for type 1 diabetes.

Iakovliev et al., 2023, The American Journal of Human Genetics *110*, 913–926
June 1, 2023 © 2023 The Author(s).
https://doi.org/10.1016/j.ajhg.2023.04.003

Check for updates
CellPress

Genome-wide aggregated *trans*-effects on risk of type 1 diabetes: A test of the "omnigenic" sparse effector hypothesis of complex trait genetics

Andrii Iakovliev,[1] Stuart J. McGurnaghan,[2] Caroline Hayward,[2] Marco Colombo,[3] Debby Lipschutz,[2] Athina Spiliopoulou,[1] Helen M. Colhoun,[2] and Paul M. McKeigue[1,*]

Summary

The "omnigenic" hypothesis postulates that the polygenic effects of common SNPs on a typical complex trait are mediated through *trans*-effects on expression of a relatively sparse set of effector ("core") genes. We tested this hypothesis in a study of 4,964 cases of type 1 diabetes (T1D) and 7,497 controls by using summary statistics to calculate aggregated (excluding the HLA region) *trans*-scores for gene expression in blood. From associations of T1D with aggregated *trans*-scores, nine putative core genes were identified, of which three—*STAT1*, *CTLA4* and *FOXP3*—are genes in which variants cause monogenic forms of autoimmune diabetes. Seven of these genes affect the activity of regulatory T cells, and two are involved in immune responses to microbial lipids. Four T1D-associated genomic regions could be identified as master regulators via *trans*-effects on gene expression. These results support the sparse effector hypothesis and reshape our understanding of the genetic architecture of T1D.

Introduction

The "omnigenic" hypothesis postulates that most of the genetic effects on a typical complex trait are mediated through weak *trans*-effects of common variants that coalesce on expression of a relatively sparse set of "core" effector genes in relevant tissues.[1] The rationale for this hypothesis is based on two established findings: (1) for a typical complex trait, most of the heritability is accounted for by small effects of many common variants; (2) for most genes, about 70% of the heritability of expression is attributable to *trans*-acting variants of small effect.[2] Genes that have *trans*-effects on expression of multiple core genes are termed "peripheral master regulators."[3]

As *cis*-expression quantitative trait loci (*cis*-eQTLs) usually have much larger effect sizes than *trans*-eQTLs, testing one SNP at a time for association of a disease with variants influencing gene expression will detect mostly *cis*-effects. If gene expression affects disease risk but most of the heritability of expression is attributable to *trans*-effects, the associations of the disease with aggregated effects of *trans*-acting variants on expression of the gene should generally be stronger than the associations with *cis*-acting variants in that gene. Thus, it may be possible to identify core genes by using summary results of genome-wide association studies (GWASs) of gene expression in relevant tissues to aggregate the *trans*-effects on expression of each gene as a genome-wide *trans*-score, then testing these *trans*-only genotypic scores, one gene at a time, for association with the disease.

For a disease-associated target gene that is identified only through *trans*-effects, any evidence of a *cis*-effect provides independent validation. Such evidence may come from association of disease with a *cis*-eQTL score for the target gene or simply with SNPs in or near the target gene. The most compelling evidence for causality is if the target gene identified through *trans*-effects on expression is a monogenic cause of the disease under study.

A practical limitation to applying this approach to investigating the genetic architecture of a complex trait is that GWAS results based on very large samples are required to learn small *trans*-effects on gene expression, which are then aggregated in genome-wide *trans*- scores. For gene expression in whole blood it is now feasible to construct such *trans*-scores, as results of a meta-analysis based on 31,684 individuals are now available from the eQTLGen Consortium.[4] This approach may be especially applicable to autoimmune diseases such as type 1 diabetes (T1D) because leukocytes in blood are a relevant tissue in which to study effects on gene expression. About half the genetic information for discrimination in T1D (defined as the logarithm of the sibling recurrence risk ratio) is accounted for by the HLA region[5] and about one-quarter by the top 40 SNPs outside the HLA region.[6] Thus, although the genetic architecture of T1D is less polygenic than that of most other complex traits, there is still a substantial contribution from polygenic effects.

The objective of this study was to test whether the omnigenic model applies to T1D with a large case-control dataset. We sought to determine whether putative core genes could be identified from associations of T1D with

[1]Usher Institute, College of Medicine and Veterinary Medicine, University of Edinburgh, Teviot Place, Edinburgh EH8 9AG, Scotland; [2]Institute of Genetics and Cancer, College of Medicine and Veterinary Medicine, University of Edinburgh, Western General Hospital Campus, Crewe Road, Edinburgh EH4 2XUC, Scotland; [3]University of Leipzig, Medical Faculty, University Hospital for Children and Adolescents, Center for Pediatric Research, Leipzig, Germany
*Correspondence: paul.mckeigue@ed.ac.uk
https://doi.org/10.1016/j.ajhg.2023.04.003.
© 2023 The Author(s). This is an open access article under the CC BY license (http://creativecommons.org/licenses/by/4.0/).

genome-wide *trans*-scores computed by aggregating the effects of multiple *trans*-eQTLs or *trans*-QTLs for circulating protein levels (*trans*-pQTLs). We describe also a conventional SNP-by-SNP GWAS analysis and the results of using *cis*-eQTL and *cis*-pQTL scores to investigate possible mediators of SNP associations with T1D that have been reported previously.

Subjects and methods

The study was carried out in accordance with the ethical principles in the Declaration of Helsinki and was approved by the Tayside Research Ethics Committee (reference 10/S1402/43). Informed consent was obtained from all participants.

Cases and controls

The Scottish Diabetes Research Network Type 1 Bioresource (SDRNT1BIO) is a consented cohort of 6,127 people clinically diagnosed as having T1D and aged 16 years and older at recruitment between 2010 and 2013. It comprises one-third of the adult population with T1D in Scotland.[7] Questionnaire data and entry day samples from this cohort were linked to clinical data from the Scottish Care Information Diabetes Collaboration electronic health records. The cases were restricted to those with definite T1D, defined as at least two insulin prescriptions within 1 year of diagnosis and no reported diagnosis of monogenic diabetes on questionnaire. Those who at entry had plasma C-peptide greater than 600 pmol/L and were negative for three auto-antibodies (glutamic acid decarboxylase, tyrosine phosphatase-related islet antigen 2, and zinc transporter 8) were excluded as "possible type 2," as described previously.[8] The controls were participants in the Generation Scotland Family Health Study, a family-based cohort of 24,000 volunteers across Scotland aged 18 years and older at recruitment between 2006 and 2011.[9] Individuals were excluded if they had any record of diabetes based on self report, linkage to hospital records, or any prescription of insulin or antidiabetic oral medications.

Genotyping

The SDRNT1BIO cohort was genotyped with the Illumina Human Core Exome 24 bead array in the Center for Public Health Genomics lab in the University of Virginia. The Generation Scotland cohort was genotyped with the Illumina OmniExpressExome 8V 1-2A bead array. We applied a standard quality control (QC) pipeline to the allele signals including SNP calling with zCall,[10] alignment to human genome build hg19 (GRCh37) and filtering of SNPs to exclude those with minor allele frequency less than 1%, those that were monomorphic in the 1000 Genomes[11] and UK10K[12] reference panels, and those with genotype frequencies deviant from Hardy-Weinberg equilibrium ($p < 10^{-10}$ for non-HLA regions and $p < 10^{-12}$ for the HLA region).

Genotypes from case and control cohorts were combined and checked for heterozygosity and sex concordance. After pruning to remove SNPs in linkage disequilibrium, we calculated the genotype relationship matrix (correlation between genotype vectors). We performed pruning to remove related individuals until there were no remaining pairs of individuals with genotype correlation > 0.05. Principal components were calculated across the remaining individuals. The genotypes were phased with the Eagle[13] and ShapeIt (duoHMM)[14] software packages. We performed imputation against UK10K and 1000 Genomes reference panels by using the Sanger Institute's online imputation service. SNP positions were lifted to genome build hg38 (GRCh38) for all analyses after this step.

Statistical analyses

For the SNP-by-SNP GWAS, we tested each SNP for association with T1D in a logistic regression model adjusted for sex, age, and the first three genotypic principal components by using SNPTEST[15] with imputed genotype probabilities. SNPs with minor allele frequency less than 0.5% or imputed information content less than 70% (as calculated by SNPTEST) were excluded. We annotated the results with genomic regions previously reported to be associated with T1D. These were based on Robertson et al. (2021).[16] This dataset has 224 hit regions, of which 163 are outside the HLA region. The candidate gene assigned for each hit region is that given in the original tables from the Type 1 Diabetes Knowledge Portal (https://t1d.hugeamp.org/), Supplementary Table 9 (dominant/recessive models) of Robertson et al. (2021),[16] and Supplementary Table 5 of Vujkovic et al. (2020).[17] The start and end positions of the transcription site of each gene (not including the promoter region) were obtained from Ensembl.[18]

We next constructed scores for aggregated *trans*-effects on gene expression and tested for association with T1D. For gene expression in whole blood, we used summary GWAS statistics obtained from the eQTLGen meta-analysis[4] for 17,422 genes. In that study only 10,317 trait-associated SNPs, identified from GWAS Catalog and Immunobase, were tested by eQTLGen for *trans*-association with gene expression. We used the GENOSCORES platform[19] to calculate genome-wide *trans*-scores for expression of each gene and circulating levels of each protein as follows.

1. Summary statistics were filtered at $p < 10^{-5}$. For each clump of variants containing at least one SNP with $p < 10^{-6}$ and separated by at least 1 Mb from other such clumps, regression coefficients for all SNPs in that clump were included in a locus-specific weights vector. This threshold is less stringent than would be used for declaring association in a genome-wide study, as our objective was to construct predictors rather than to test for association.
2. For each clump of SNPs, the correlations between genotypes for the retained variants were extracted from the European ancestry subset of the 1000 Genomes panel. Each locus-specific weights vector was premultiplied by the inverse of this correlation matrix to adjust for linkage disequilibrium. This adjustment approximates the regression coefficients that would be obtained in a multiple regression analysis of the individual-level GWAS dataset. The locus-specific score was calculated for each individual as the dot product of the individual's genotypes and the adjusted weights vector.
3. Each locus-specific score was classified as *cis*- if the distance from the clump to the transcription site of the respective gene was less than or equal to 50 kb, *cis-x* if the distance was 50 kb to 5 Mb, and *trans*- if the distance was more than 5 Mb. The genome-wide *trans*-score was computed as the sum of locus-specific *trans*-scores for the target gene. As the HLA region is a hotspot for *trans*-eQTLs for genes involved in the immune system,[20] and associations of these *trans*-eQTLs with T1D are heavily confounded by the direct

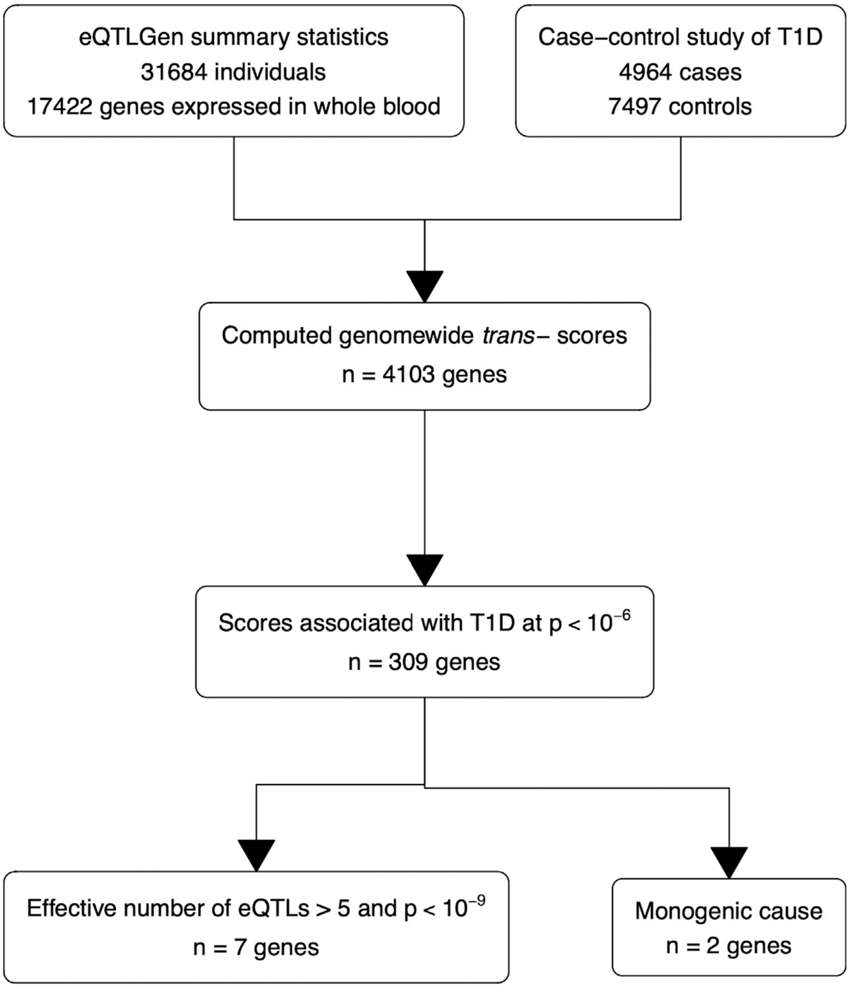

Figure 1. Flow chart of selection of putative effector genes
The genes were identified using T1D association with genome-wide *trans*-scores, locus diversity, and whether the variation in the gene is a known monogenic cause of T1D.

effects of HLA antigens on T1D risk as described below, the HLA region (from 25 to 34 Mb on chromosome 6) was excluded from the computation of genome-wide *trans*-scores. This procedure generated 4,824 *cis*-scores for 4,702 genes and 4,102 genome-wide *trans*-scores.

Each of these scores was tested for association with T1D in a logistic regression model with T1D as dependent variable and the first three genotypic principal components as covariates. All scores were scaled to have standard deviation of 1 so that the log odds ratios represent the difference in log odds associated with a difference of 1 standard deviation in the score. The information for discrimination, in natural log units, can be calculated as half the square of this standardized log odds ratio.[21]

Where SNPs that are associated with T1D have pleiotropic *trans*-effects on gene expression, we expect genome-wide *trans*-scores for these genes to be associated with T1D even if they are only "bystander" genes that are not in the causal path from SNPs to T1D. Where a *trans*-score for expression of a gene is associated with T1D, that gene is more likely to be a core gene if the score is the sum of multiple locus-specific *trans*-scores. This is because aggregating effects of multiple *trans*-eQTLs on a target gene will amplify the signal of a core gene but not the noise generated by *trans*-effects on the expression of a "bystander" gene. As an index of the effective number of unlinked *trans*-eQTLs contributing to each genome-wide *trans*-score, we calculated the diversity index or Hill number.[22] For each gene, the diversity index was computed from the variances $\sigma_1, \ldots, \sigma_K$ of the K locus-specific *trans*-scores as $2^{-\sum p_i \log_2 p_i}$, where $p_i = \sigma_i^2 / \sum \sigma_i^2$. This index can take values from 1, if one of the eQTLs has much larger variance than the others, to K, if the variances of the locus-specific *trans*-scores are equal. Figure 1 shows a flow chart of this process for selection of putative effector genes.

The procedure described above for effects on gene expression was repeated for effects on circulating proteins. We extracted summary GWAS statistics from five studies of circulating proteins: 48 proteins in plasma or serum on the Bio-Rad cytokine panel,[23] 1,478 proteins in plasma on the SomaLogic panel,[24] 4,719 proteins in plasma on the SomaScan v4 panel,[25] 83 proteins in plasma on the Olink cardiovascular panel,[26] and 70 proteins in plasma on the Olink inflammation panel.[27] We computed *cis*-scores for 889 proteins and *trans*-scores for 2,745 proteins. The aggregation of locus-specific *trans*-scores into genome-wide *trans*-scores was performed in the same manner as for eQTLs.

For putative core genes identified by this procedure, we tested for interaction with the effects of the HLA region on T1D risk by using a case-only test of association between the *trans*-score and the risk score for the HLA region. We constructed summary risk scores for the HLA region on the basis of eight variables derived from five SNPs that tag class I and class II haplotypes as described by Oram et al.[28] The weights for these eight variables were learned by fitting a multiple logistic regression model to the case-control study.

To test whether genetic effects on the frequencies of immune cell types could explain the associations of T1D with genome-wide *trans*-scores for gene expression, we tested for association of T1D with genome-wide scores for immune cell phenotypes. We used summary statistics from two studies of immune cell phenotypes in peripheral blood[29,30] with sample sizes of 497 and 1,629 individuals to calculate genome-wide scores for each immune cell phenotype and tested these scores for association with T1D.

Results

SNP associations with T1D

We first undertook a conventional SNP-by-SNP GWAS analysis of this case-control study. Figure 2A shows a Manhattan plot of the SNP associations with T1D.

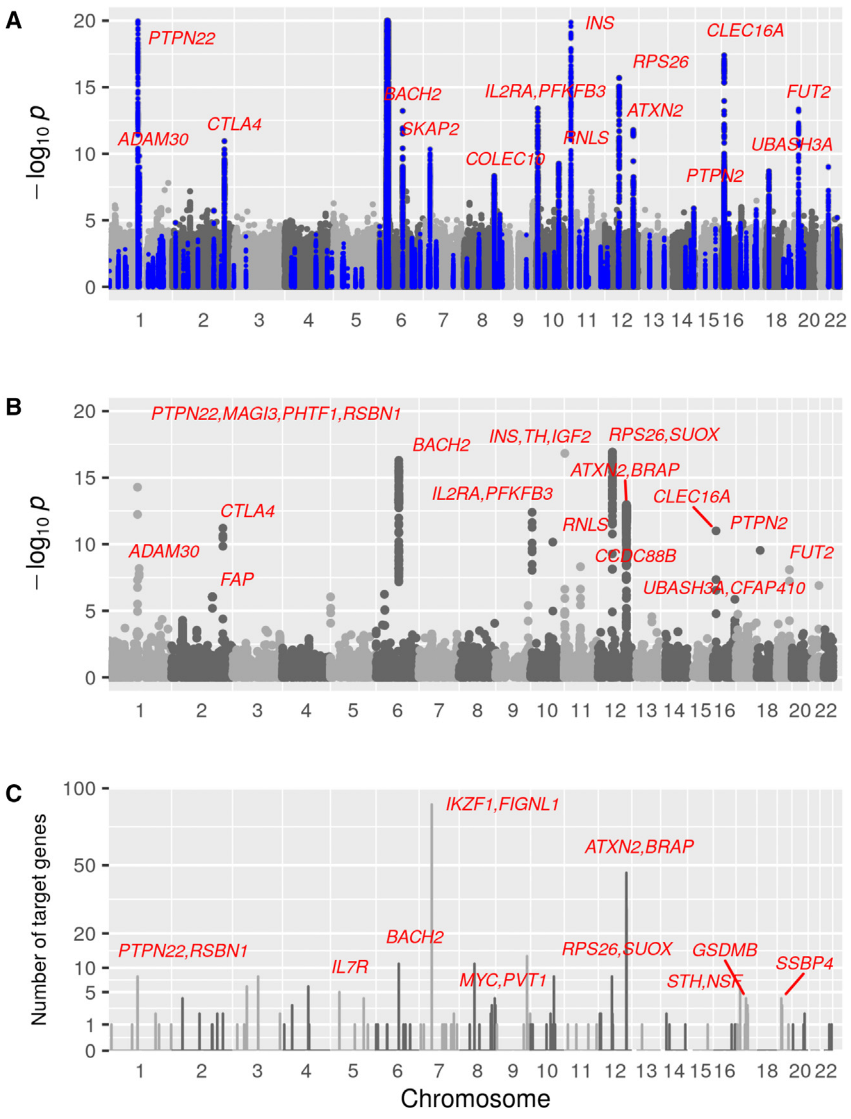

Figure 2. Manhattan plots for SNPs, locus-specific *trans*-scores, and counts of *trans*-scores originating from each locus

(A) Manhattan plot of SNP associations with T1D in case-control study. Minus $\log_{10}$ p value truncated at 20. SNPs within clumps of T1D-associated SNPs reported by the Type 1 Diabetes Knowledge Portal are highlighted in blue. A clump is defined by flanking regions of 20 kb if a single SNP is reported. Each clump with lead SNP ($-\log_{10} p > 6$) is labeled with the nearest T1D-associated gene.

(B) Manhattan-like plot of T1D associations with locus-specific *trans*-scores, excluding the HLA region. Scores for which $-\log_{10} p > 6$ are labeled as in (A).

(C) Number of *trans*-eQTLs that each genomic region contributes to T1D-associated genome-wide *trans*-scores. Each set of overlapping SNP clumps contributing to T1D-associated *trans*-scores defines a region. Regions contributing to more than two scores are labeled with previously reported T1D-associated genes.

Clumps of SNPs documented in the Type 1 Diabetes Knowledge Portal as containing T1D-associated SNPs are highlighted. Of the regions detected at genome-wide significance in this study, two have not previously been described in detail: the *ADAM30* region on chromosome 1 and the *COLEC10* region on chromosome 8. The top SNP in the *ADAM30* region was rs406767 at 120.01 Mb, within the *NOTCH2* transcription site. T1D was associated with the *cis*-eQTL score for expression of *NOTCH2* in whole blood (standardized odds ratio 0.94, $p = 5 \times 10^{-4}$) and with the *cis*-pQTL score for *REG4* (standardized odds ratio 0.92, $p = 1 \times 10^{-5}$). The top T1D-associated SNP in the region labeled *COLEC10* is at 119.05 Mb within *COLEC10*. The only *cis*-QTL score associated with T1D in this region is a pQTL score for *TNFRSF11B* (transcription site 119.79 to 119.81 Mb, standardized odds ratio 0.92, $p = 2 \times 10^{-5}$).

To investigate whether *cis*-effects on gene expression or protein levels could help with identifying the local genes mediating the effects of T1D-associated SNPs, we tested all *cis*-eQTL and *cis*-pQTL scores for association with T1D, excluding scores for genes within the HLA region. Of 4,801 *cis*-eQTL scores and 1,003 *cis*-pQTL scores tested, 11 were associated with T1D at $p < 10^{-6}$, as shown in Table 1. All these T1D-associated *cis*-eQTLs were in regions where T1D-associated SNPs have previously been reported, but the *cis*-eQTL target was not always the same gene as that to which the SNP association in that region had been attributed.

- The association of T1D with SNPs in the 12q13 region has been attributed to *ERBB3*.[31,32] Table S1 shows however that the strongest *cis*-QTL score associations with T1D in this region were for *RAB5B*, *ERBB3*, and *IL-23A*.
- The association of T1D with SNPs in the 12q24.12 region (111.3–111.9 Mb) containing *ATXN2* has been attributed to a non-synonymous SNP in *SHB23*.[31] The T1D-associated SNPs in the 12q24 region lie in a range from 111.39 to 112.5 Mb, corresponding to a linkage disequilibrium block in European populations. Tables 1 and S1 show, however, that the transcription sites of the genes with T1D-associated *cis*-eQTL or *cis*-pQTL scores extend over a broader region from 108.56 to 114.35 Mb (bands 12q24.11 to 12q24.13), including *ISCU*, *SELPLG*, *PPTC7*, *GLTP*, *TRAFD1*, *SDSL* and *TBX5*.

T1D was strongly associated with an extended *cis*-pQTL score for *NECTIN2* generated by SNPs from 48.59 to 48.74

Table 1. Genes for which *cis*-eQTL or *cis*-pQTL score is associated with T1D at $p < 10^{-6}$

	Transcription site		*cis*-eQTL score		*cis*-pQTL score		
Gene symbol	Chr	Start position (Mb)	Log odds ratio	p value	Log odds ratio	p value	T1D SNP association within 200 kb of transcription site
PHTF1	1	113.70	−0.14	5×10^{-13}	–	–	*PTPN22*, *MAGI3*, *PHTF1*, *RSBN1*
BACH2	6	89.93	−0.13	2×10^{-11}	–	–	*BACH2*
CCDC88B	11	64.34	0.11	7×10^{-9}	–	–	*CCDC88B*
RAB5B	12	55.97	0.14	9×10^{-14}	–	–	*RPS26*, *SUOX*
ERBB3	12	56.08	.	.	−0.14	6×10^{-13}	–
IL23A	12	56.33	−0.16	2×10^{-17}	–	–	*RPS26*
ISCU	12	108.56	−0.01	0.4	0.14	1×10^{-12}	–
SELPLG	12	108.62	0.02	0.3	0.11	2×10^{-9}	–
GLTP	12	109.85	–	–	0.11	6×10^{-9}	–
PPTC7	12	110.53	−0.14	4×10^{-13}	–	–	*ATXN2*
ALDH2	12	111.77	0.01	0.5	0.10	1×10^{-7}	*ATXN2*, *BRAP*
TRAFD1	12	112.13	0.11	4×10^{-8}	–	–	*ATXN2*, *BRAP*
SDSL	12	113.42	0.11	2×10^{-9}	0.03	0.2	–
TBX5	12	114.35	–	–	0.12	1×10^{-9}	–
PLAUR	19	43.65	0.01	0.5	0.13	1×10^{-11}	–
NECTIN2	19	44.85	−0.02	0.2	−0.12	6×10^{-11}	–
KLK13	19	51.06	–	–	−0.10	3×10^{-7}	–
UBASH3A	21	42.40	−0.10	1×10^{-7}	–	–	*UBASH3A*

The *cis*-pQTL for *NECTIN2* is at 48.59 to 48.74 Mb, 3.7 Mb downstream of the transcription site and contains *FUT2*.

Mb on chromosome 19, 3.7 Mb downstream of the transcription site and spanning *FUT2*. Whether this should be classified as an extended *cis*-pQTL is uncertain, as the 3D Genome Browser[33] (see web resources) shows no Hi-C interactions between the pQTL and the transcript region in a relevant cell line (Liver-STL011). This extended *cis*-pQTL was also a *trans*-pQTL for *CCL15* (Table S6), contributing to the aggregated *trans*-score for this chemokine, which was associated with T1D as discussed below.

Associations of T1D with genome-wide *trans*-scores for gene expression

We next tested for associations of T1D with *trans*-scores for gene expression. By far the strongest associations were those generated by *trans*-eQTLs in the HLA region (Table S2). Of the 1,041 genes for which *trans*-scores could be calculated from SNPs in this region, 181 were associated with T1D at $p < 10^{-6}$. These genes include the *TRAV* and *TRBV* gene families that encode T cell receptors that recognize the antigens encoded by HLA genes. These associations of T1D with HLA region-wide *trans*-scores are so heavily confounded by the direct effects of HLA antigens on T1D risk that they are difficult to interpret. To control this confounding, the HLA region was excluded from the aggregation of locus-specific *trans*-scores into genome-wide scores.

We next tested all 4,103 genome-wide *trans*-scores for association with T1D. Of these, 309 were associated at $p < 10^{-6}$ (Table S3). Figure 2B shows that 44 regions contributed eQTLs to the 219 genome-wide *trans*-scores that were associated with T1D at $p < 10^{-9}$. Of the 32 regions contributing to two or more of these 219 T1D-associated genome-wide scores, ten contained SNPs previously reported as GWAS hits for T1D. Four regions containing T1D-associated SNPs contributed to more than five of these scores: *PTPN22* on chromosome 1, *BACH2* on chromosome 5, *IKZF1* on chromosome 7, and the 12q24 region containing *ATXN2*.

For further analysis of the genes with strongest evidence for a causal relationship of function to T1D, the T1D-associated genome-wide *trans*-scores were restricted to those that met the following criteria: (1) effective number of eQTLs greater than 5 and $p < 10^{-9}$ or (2) monogenic cause of autoimmune diabetes and $p < 10^{-6}$. Seven putative core genes were designated on the basis of criterion (1); *STAT1* and *FOXP3* were designated as putative core genes on the basis of criterion (2).

Table 2 summarizes the associations of T1D with the nine genes designated as putative core genes. Three of these genes—*CTLA4*, *STAT1*, and *FOXP3*—have been previously identified as causing monogenic autoimmune

Table 2. Candidates for core gene status

Gene symbol	Transcription site: Chr	Transcription site: Start position (Mb)	*trans*-score: Effective number of eQTLs	*trans*-score: Log odds ratio	*trans*-score: p value	*cis*-score: Log odds ratio	*cis*-score: p value	T1D SNP associations within 200 kb of transcription site	Monogenic diabetes
**Effective number of eQTLs > 5 and $p < 10^{-9}$**									
CD1E	1	158.35	6.5	−0.12	8×10^{-10}	−0.02	0.3	–	–
CD247	1	167.43	5.8	−0.13	2×10^{-11}	0.02	0.4	–	–
CTLA4	2	203.87	7.6	0.21	2×10^{-28}	−0.08	3×10^{-5}	*CTLA4*	+
CD5	11	61.10	5.5	0.20	7×10^{-28}	−0.01	0.5	*SLC15A3*	–
IL10RA	11	117.99	5.7	0.17	9×10^{-20}	−0.02	0.2	–	–
MEOX1	17	43.64	5.5	−0.14	2×10^{-13}	0.02	0.2	–	–
LGALS3BP	17	78.97	5.3	0.15	7×10^{-14}	0.00	0.8	–	–
**Monogenic cause of autoimmune diabetes and** $p < 10^{-6}$									
STAT1	2	190.91	5.7	0.10	9×10^{-8}	−0.02	0.2	*STAT4*	+
FOXP3	X	49.25	2.9	0.23	5×10^{-35}	–	–	–	+

Criteria for including genes based on association of T1D with *trans*-score for expression are shown in the header rows. The association of T1D with the *cis*-score for that gene is shown if a *cis*-score was constructed and was associated with T1D at $p < 0.001$. Any SNP association reported by the Type 1 Diabetes Knowledge Consortium is shown if the top SNP in the clump was within 200 kb of the transcription site of the target gene. This criterion excludes a clump of T1D-associated SNPs containing *UBE4A*, 263 kb downstream of *IL10RA*. The gene for the SNP association is that identified as the candidate or nearest gene in the original study.

diabetes.[34] For four of these genes—*CTLA4*, *STAT1*, *CD5*, and *IL10RA*—T1D associations with SNPs in or near the gene have been reported by the Type 1 Diabetes Knowledge Consortium. For *CTLA4*, both the *cis*-score and the genome-wide *trans*-score for gene expression were associated with T1D. Although the *cis*-effect is in the opposite direction to the *trans*-effect, this is explicable as the T1D-associated SNPs in *CTLA4* alter the splicing of the transcript.[35,36]

To investigate the specificity of the associations of genome-wide *trans*-scores with T1D, we examined the correlations between these scores in the control group. Figure S1 shows that the *trans*-scores for the putative core genes were not highly correlated with each other. The only other gene with a *trans*-score that was highly correlated ($r^2 > 0.7$) with a *trans*-score for any of the putative core genes was *PARP3*, which was highly correlated ($r = 0.86$) with the score for *LGALS3BP*. Table 3 shows that 14 *trans*-eQTLs within 200 kb of clusters of SNPs that have been reported as T1D associated contributed to one or more of the genome-wide *trans*-scores for these nine putative core genes. Figures 3 and 4 show that the *trans*-eQTLs contributing to the genome-wide scores for these nine genes include some loci that were weakly associated with T1D but did not reach genome-wide significance in this study or in previously reported studies.

To investigate whether these associations of T1D with genome-wide *trans*-scores could be explained by variation in the profile of immune cell types, we tested for association of T1D with genome-wide scores for immune cell phenotypes. Tables S4 and S5 show that T1D was not associated with genome-wide scores for any immune cell phenotypes, including the levels of regulatory T cells as percentages of T cells and of $CD4^+$ T cells.

Associations of T1D with genome-wide *trans*-scores for circulating protein levels

We repeated the procedure for analysis of aggregated *trans*-effects by using the scores for *trans*-effects on circulating protein levels. This yielded five putative core genes—*EIF4G3*, *CCL19*, *CRTAM*, *LIN7B* and *NCR1*—on the basis of association of T1D at $p < 10^{-9}$ with a *trans*-score for the gene product and effective number of *trans*-pQTLs > 5 and another nine—*CD5L*, *CD48*, *FCGR3B*, *GCG*, *CXCL9*, *LAG3*, *CCL15*, *ICAM2*, and *BPIFA2*—at the less stringent threshold of $p < 10^{-6}$ (Table 4). Of these, only *GCG*, which encodes glucagon, has been previously reported as T1D associated in a conventional SNP-by-SNP GWAS.[16] Correlations between these scores in the control group are weak except for *EIF4G3* and *LIN7B* ($r = 0.74$). For *LAG3*, the expression score (Table S3) also was strongly associated with T1D ($p = 7 \times 10^{-10}$), but as the effective number of QTLs for the expression score was only 2.9, it was not included in Table 2 as a putative core gene.

Table S6 shows that many of the *trans*-pQTLs contributing to the T1D-associated genome-wide *trans*-scores for these proteins overlapped with regions in which SNP associations with T1D have previously been reported. Thus, the *trans*-pQTLs for circulating CXCL9 (chemokine ligand 9) levels included SNPs within 200 kb of the transcription sites of *PTPN22*, *BCL11A*, *CCR9*, *KIAA1109*, *TULP1*, *INS*, *ATXN2*, *GPR183*, and *UBASH3A*. All these genes are recorded by the Type 1 Diabetes Knowledge Consortium as associated with T1D.

Table 3. T1D-associated *trans*-eQTL regions that contribute to *trans*-scores for putative core genes

Chr	Start position (Mb)	End position (Mb)	Genes to which T1D association with SNPs within 200 kb of the *trans*-eQTL region was attributed	Target genes that are putative core genes
1	113.63	113.83	*PTPN22, MAGI3, PHTF1, RSBN1*	*CD5, CTLA4, FOXP3, CD247, IL10RA*
1	199.04	199.04	*PTPRC*	*CD1E*
2	43.33	43.49	*PLEKHH2*	*CD1E, IL10RA*
2	203.88	203.88	*CTLA4*	*MEOX1*
5	35.84	35.94	*IL7R*	*CTLA4, MEOX1, FOXP3*
6	0.41	0.42	*IRF4*	*CTLA4*
6	90.10	90.32	*BACH2*	*CD1E, MEOX1, CTLA4*
10	6.05	6.07	*FBXO18, IL2RA, PFKFB3, DKFZP667F0711*	*CTLA4*
11	128.53	128.54	*FLI1*	*MEOX1*
12	56.00	56.00	*RPS26, SUOX*	*CD5*
12	110.90	112.66	*ATXN2, BRAP*	*STAT1, LGALS3BP*
14	98.02	98.03	*C14orf64*	*CD5*
17	45.44	45.83	*STH*	*LGALS3BP*
17	46.71	46.78	*STH, NSF*	*LGALS3BP*

trans-eQTL regions are shown if they are within 200 kb of a clump of T1D-associated SNPs and contribute to one or more *trans*-scores for the putative core genes in Table 2.

Discussion

On the basis of aggregating the effects of multiple *trans*-eQTLs, this study identifies nine top candidates as putative core genes for T1D. *Trans*-eQTL effects of T1D-associated SNPs on *STAT1* have been noted previously.[4,37] Although four of these are validated by evidence of *cis*-effects as defined above, the only one that would have been identified as a core gene for T1D in a conventional SNP-by-SNP GWAS analysis is *CTLA4*. Of these putative core genes, seven are involved in induction and activity of CD4$^+$ regulatory T cells (Tregs). *FOXP3* is the canonical marker and regulator of Tregs.[38] *CTLA4* is expressed on Tregs where it inhibits CD28-mediated activation of T cells.[39] *STAT1* mediates the inhibition by IFNγ of induction of Tregs[40]; gain-of-function mutations in *STAT1* cause autoimmune diabetes. *CD5* promotes induction of Tregs by blocking mTOR activation.[41] *IL10RA* activates *STAT3* in Tregs, which suppresses T_h17 inflammatory responses.[42] SNPs in *IL10*, which encodes the ligand, are associated with T1D. *MEOX1* overexpression reprograms Tregs to acquire a transcriptional profile associated with tumor infiltration.[43] *CD247* encodes the CD3ζ chain subunit of the T cell receptor complex. In congenic strains derived from the NOD mouse model, the NOD allele at *Cd247* is associated with lower expression of CTLA-4 in Tregs and with higher rates of autoimmune diabetes.[44]

The other two putative core genes are involved in the innate and acquired immune response to lipids (usually of microbial origin). *LGALS3BP* inhibits transforming growth factor β-activated kinase 1-dependent activation of nuclear factor-κB by lipopolysaccharides.[45] *CD1E* is expressed intracellularly, where it modulates the loading of lipid antigens onto other CD1 isotypes that are displayed at the cell surface.[46]

As T1D was not associated with genotypic scores for the absolute or relative levels of Tregs or with scores for other immune cell phenotypes, it is unlikely that the associations of T1D with *trans*-scores for genes such as *FOXP3* that are expressed by Tregs can be explained by effects on the abundance of this cell type, although this cannot be ruled out, as the summary statistics we used to calculate scores for immune cell phenotypes were based on studies with relatively modest sample sizes.

The proportion of non-HLA heritability of T1D that is accounted for by the *trans*-scores in Table 2 is modest. For the *FOXP3* *trans*-score, for instance, the information for discrimination (calculated as half the square of the standardized log odds ratio) is 0.026 natural log units, which is about 2% of the non-HLA genetic information for discrimination for T1D, assuming that the total genetic information for discrimination is about 2.5 (equivalent to sibling recurrence risk ratio of 12) and that half of this is contributed by genes outside the HLA region. The current version of the eQTLGen meta-analysis tested only 10,317 SNPs previously identified as trait-associated for *trans*-association with gene expression. As this will have missed many *trans*-eQTLs, our study is likely to underestimate the contribution of core genes to T1D.

Another limitation of the current version of the eQTLGen dataset is that the gene expression assays do not

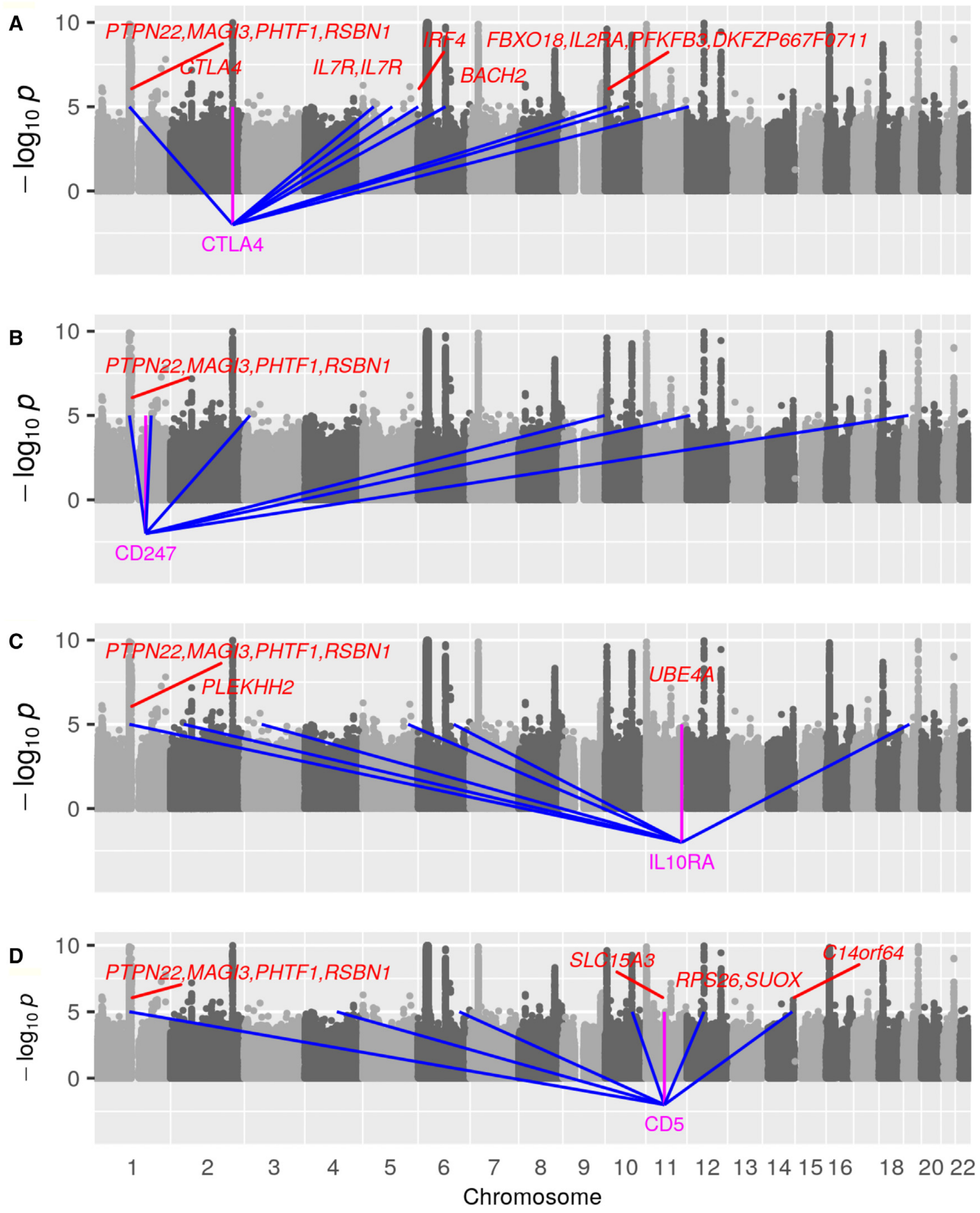

Figure 3. *Trans*-eQTLs contributing to *trans*-scores for *CTLA4, CD247, IL10RA,* and *CD5*
(A–D) eQTLs for the first four putative core genes in Table 2, *CTLA4* (A), *CD247* (B), *IL10RA* (C), and *CD5* (D), are overlaid on the Manhattan plot of SNP associations with T1D. The *cis*-eQTL for each core gene is indicated with a magenta line, and *trans*-eQTLs contributing to the genome-wide *trans*-score for each core gene are indicated with blue lines. The T1D-associated regions reported by Type 1 Diabetes Knowledge Portal are labeled in red where they overlap with the loci contributing to the genome-wide *trans*-scores.

necessarily distinguish between splicing variants that may be differentially regulated by *trans*-eQTLs. Both *FOXP3*[47] and *CTLA4*[35,36] are expressed in alternatively spliced isoforms that have different functional effects: this may explain why the *trans*-score associations of these genes with T1D are in the opposite direction to that expected given that loss-of-function mutations in these genes cause autoimmune diabetes.

The overlap of *trans*-eQTLs for these putative core genes with regions in which SNP associations with T1D have been reported suggests that some SNP associations that have no obvious interpretation in terms of *cis*-effects,

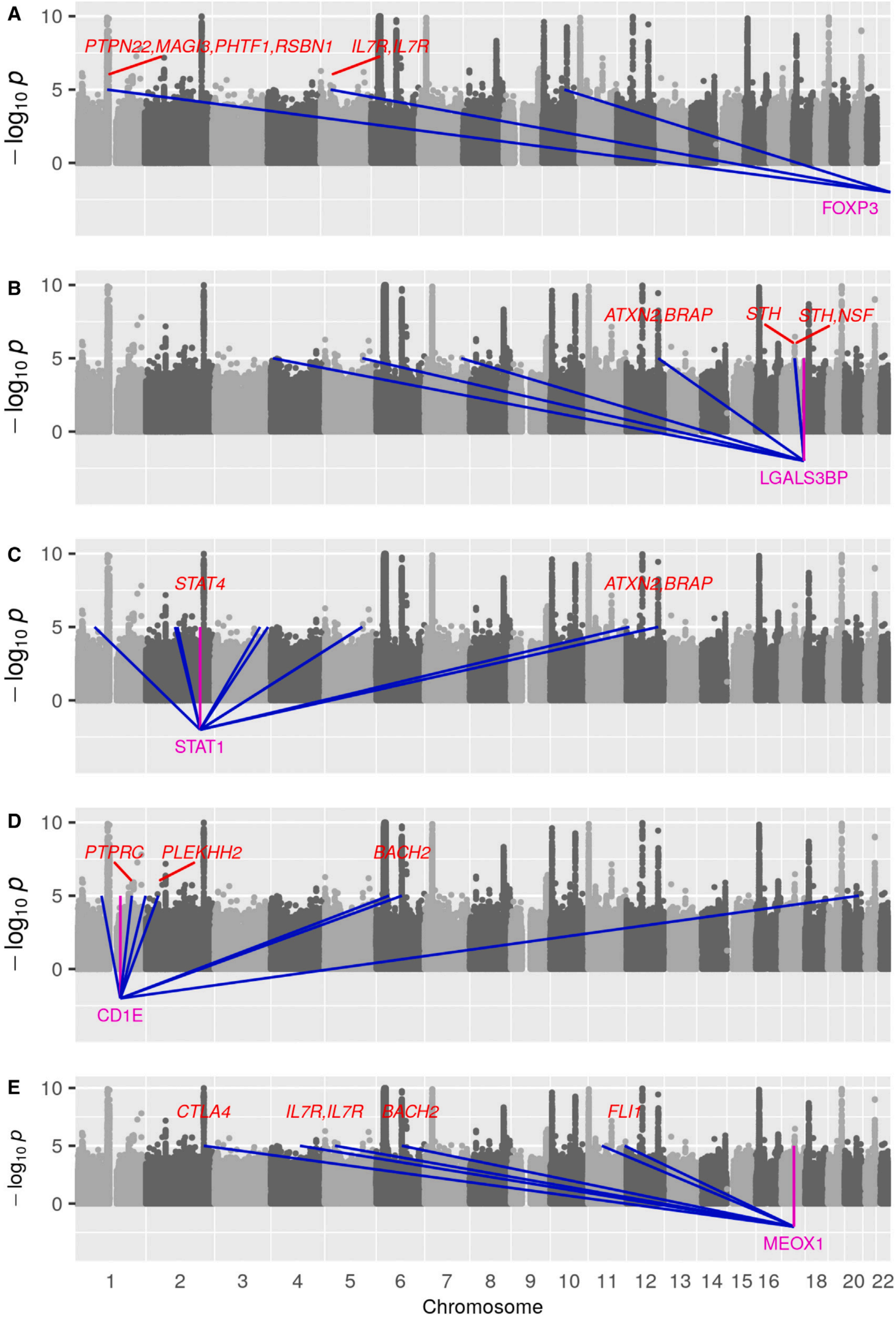

Figure 4. *Trans*-eQTLs contributing to *trans*-scores for *FOXP3*, *LGALS3BP*, *STAT1*, *CD1E*, and *MEOX1*
(A–E) eQTLs for the last five putative core genes in Table 2, *FOXP3* (A), *LGALS3BP* (B), *STAT1* (C), *CD1E* (D), and *MEOX1* (E), are overlaid on the Manhattan plot of SNP associations with T1D. The *cis*-eQTL for each core gene is indicated with a magenta line, and *trans*-eQTLs contributing to the genome-wide *trans*-score for each core gene are indicated with blue lines. The T1D-associated regions reported by the Type 1 Diabetes Knowledge Portal are labeled in red where they overlap with the loci contributing to the genome-wide *trans*-scores.

Table 4. **Candidates for core gene status based on *trans*-effects on protein levels**

		Transcription site		*trans*-score			*cis*-score	
**Protein name**	**Gene encoding protein**	**Chr**	**Start position (Mb)**	**Effective number of pQTLs**	**Log odds ratio**	**p value**	**Log odds ratio**	**p value**
Eukaryotic translation initiation factor 4 gamma 3	*EIF4G3*	1	20.81	13.0	−0.12	1×10^{-10}	–	–
CD5 antigen-like	*CD5L*	1	157.83	15.7	0.10	6×10^{-8}	−0.01	0.6
CD48 antigen	*CD48*	1	160.68	10.0	0.10	7×10^{-8}	−0.03	0.2
Low affinity immunoglobulin gamma Fc region receptor III-B	*FCGR3B*	1	161.62	10.5	0.10	5×10^{-7}	0.01	0.7
Glucagon	*GCG*	2	162.14	8.0	0.09	6×10^{-7}	–	–
Monokine induced by interferon-gamma (CXCL9)	*CXCL9*	4	76.00	11.4	0.10	2×10^{-7}	−0.06	0.001
C-C motif chemokine 19	*CCL19*	9	34.69	9.4	0.16	2×10^{-16}	0.04	0.05
Cytotoxic and regulatory T cell molecule	*CRTAM*	11	122.84	9.8	0.15	1×10^{-15}	−0.02	0.3
Lymphocyte activation gene 3 protein	*LAG3*	12	6.77	16.4	0.09	8×10^{-7}	–	–
C-C motif chemokine 15	*CCL15*	17	36.00	19.9	−0.11	2×10^{-8}	−0.02	0.2
Intercellular adhesion molecule 2	*ICAM2*	17	64.00	18.0	0.10	5×10^{-7}	–	–
Protein lin-7 homolog B	*LIN7B*	19	49.11	15.9	−0.12	2×10^{-10}	–	–
Natural cytotoxicity triggering receptor 1	*NCR1*	19	54.91	7.7	0.12	1×10^{-10}	0.05	0.01
BPI fold-containing family A member 2	*BPIFA2*	20	33.16	6.0	0.10	2×10^{-7}	0.04	0.02

Genes included in this table are those with *trans*-scores that are associated with T1D at $p < 10^{-6}$, with effective number of pQTLs > 5.

such as the association of T1D with SNPs in the *PLEKHH2* and *NSF* regions, may be explicable as *trans*-effects on the expression of core genes. We can tentatively identify four regions containing “peripheral master regulators” for T1D on the basis that they contribute *trans*-effects to multiple genes for which genome-wide *trans*-scores are associated with T1D. These regions are *PTPN22*, *BACH2*, *IKZF1*, and the 12q24 region containing *ATXN2* and *SH2B3*. Not surprisingly, SNPs in these regions containing master regulators are associated with multiple autoimmune disorders.[48]

Applying the same approach to circulating proteins identifies a different set of putative core genes, most of which are biologically relevant to T1D. One of the most interesting is *NCR1*, which may underlie the tissue specificity of the autoimmune reaction in T1D. *NCR1* encodes the NKp46 receptor expressed by natural killer (NK) cells, which recognizes an unknown ligand expressed by pancreatic β cells; knockout of *Ncr1* or blocking of the gene product inhibits the development of autoimmune diabetes in the NOD mouse.[49] *CRTAM* encodes a receptor expressed on the cell surface of activated NK and $CD8^+$ T cells. In the RIP-mOVA mouse model, knockout of *Crtam* in the donor strain inhibits induction of autoimmune diabetes by exogenous $CD8^+$ T cells.[50] *LAG3* encodes an immune checkpoint receptor; deletion of this gene in the NOD mouse accelerates development of autoimmune diabetes.[51] *CXCL9*, *CCL15*, and *CCL19* encode chemokines. *CXCL9* is expressed in pancreatic β cells in response to pro-inflammatory cytokines,[52] and deletion of the gene encoding its receptor *CXCR3* accelerates diabetes in the NOD mouse.[53] *CCL15* is expressed in the intestinal mucosa as an antibacterial peptide.[54] *CCL19* is the ligand for CCR7, a receptor expressed on lymphocytes; in the NOD mouse, migration of T cells into inflamed islets is dependent on CCR7.[55] *CD48* encodes a signaling molecule that regulates innate and adaptive immune responses through binding to the CD244 receptor on NK and other cytotoxic cells.[56] *CD5L*, initially reported as an apoptosis inhibitor, binds pathogen-associated molecular patterns, suggesting a role in innate immune response.[57] *EIF4G3* regulates translation in lymph node stromal cells of genes that affect antigen presentation.[58]

The sets of putative core genes identified by eQTL and pQTL analyses do not overlap, with the exception of *LAG3*. This is not surprising, as the genes identified through eQTL analysis are mostly transcription factors or receptors expressed on the surface of immune cells, while pQTL analysis can detect only genes that affect circulating protein levels. We would not expect leukocytes in peripheral blood to contribute much to the levels of these proteins.

Although the main focus of this paper is the identification of effector genes and master regulators via *trans*-

effects, we have also used *cis*-eQTL and *cis*-pQTL associations to investigate *cis*-acting effects on T1D and to identify the genes most likely to mediate some previously reported SNP associations with T1D. For the 12q13 region containing *ERBB3*, a plausible candidate for the mediator is *IL23A*, which encodes a subunit of interleukin-23. This cytokine induces immune-mediated β cell damage and diabetes in a mouse model.[59] The association of T1D with SNPs in *FUT2* has been widely attributed to *FUT2*, as one of the top T1D-associated SNPs is a non-synonymous variant that determines ABO blood group antigen secretor status. However, no biological mechanism for the association of *FUT2* with T1D has been established. The *cis*-pQTL analysis suggests *NECTIN2*, which modulates T cell signaling by binding to the CD226 receptor, as a possible mediator. SNPs in *CD226* are associated with T1D, and deletion of this gene protects against diabetes in the NOD mouse.[60]

The approach used in this study—using summary GWAS results on gene expression to construct *trans*-only genotypic scores and then testing these for association in an individual-level study of the disease or trait of interest—complements the conventional SNP-by-SNP analysis of a GWAS, where detection of a SNP association with disease is followed by attempts to identify the gene that mediates this association. Our approach falls into the general category of transcriptome-wide association study designs. As our objective is specifically to detect effector genes through which the effects of many weak *trans*-eQTLs are mediated, we have constructed a procedure to maximize the detection of such genes on the basis of using summary-level data from a large study of gene expression to calculate genome-wide scores that aggregate the *trans*-effects on expression of each gene and then testing these scores for association with the disease under study. A related approach is "expression quantitative trait score" (eQTS) analysis in which summary-level GWAS data are used to construct genome-wide polygenic scores for traits of interest that were tested for individual-level association with gene expression in the eQTLGen dataset.[4] One limitation of the eQTS method is that it does not distinguish *trans*- and *cis*-effects on expression of each gene. A more fundamental limitation may be that testing for association of expression of each gene with a polygenic score for the disease is less statistically powerful than testing the aggregated effects of weak *trans*-eQTLs directly because any associations of disease with *trans*-eQTL effects on expression of a gene are diluted by the other SNPs included in the polygenic score. Another related approach that uses only summary statistics has been described as "aggregative *trans*-eQTL analysis," which computes canonical correlations between linear combinations of SNPs associated with expression of each gene and linear combinations of SNPs on the trait of interest.[61] A limitation of that approach is that SNP associations are coded only as present/absent: the size and direction of effects on gene expression and the trait of interest are ignored.

These results support the omnigenic hypothesis in that selecting genome-wide *trans*-scores that are generated by multiple *trans*-QTLs and are strongly associated with T1D identifies a set of genes that were mostly not detected in a conventional SNP-by-SNP analysis but are biologically highly relevant to T1D. Some of these putative effector genes are validated by *cis*-effects, by experimental models, or by effects of other genes in the same pathway. For clarity, we suggest a revision of terminology: "core genes" can include both master regulators and effector genes, and the "omnigenic" hypothesis can be more precisely denoted as the sparse effector gene hypothesis. The hypothesis has implications for how GWASs should be analyzed, suggesting that analysis should focus on identifying effector genes through aggregated *trans*-eQTL effects rather than on identifying which SNP, in a cluster of SNPs with tiny effects on the outcome, is the functional variant. Core gene effects may be more robust than SNP associations to variation between populations.[62] The method described here is readily applicable to other disorders of the immune system for which whole blood is a relevant tissue in which to study gene expression. Wider application of this approach will require availability of more comprehensive summary-level results from large GWASs of gene expression in various tissues and measurements of genetic effects on splicing variants and post-transcriptional modification.

Data and code availability

All code used in this analysis is available at https://github.com/molepi-precmed/trans-qtls. Summary-level data is available from https://doi.org/10.5281/zenodo.7786862. Accredited researchers may apply to the Public Benefit and Privacy Panel for access to the individual-level data. The application should be submitted via HSC-PBPP website https://www.informationgovernance.scot.nhs.uk/pbpphsc/. The platform used to compute locus-specific genotypic scores and the database of published GWAS summary statistics are accessible on the https://genoscores.cphs.mvm.ed.ac.uk/ platform. The corresponding R package will be shared on request. Annotation of the known T1D associations was taken from Type 1 Diabetes Knowledge Portal: https://t1d.hugeamp.org.

Supplemental information

Supplemental information can be found online at https://doi.org/10.1016/j.ajhg.2023.04.003.

Acknowledgments

This study was supported by Diabetes UK (15/0005301). The establishment of the SDRN Type 1 Bioresource was supported by the Chief Scientist Office of the Scottish Government Health Directorates (ETM/47), by Diabetes UK (10/0004010), and by in-kind contributions from the Scottish Diabetes Research Network.

Genotyping was supported by the Juvenile Diabetes Research Fund (17-2013-7). Generation Scotland received core support from the Chief Scientist Office of the Scottish Government Health Directorates (CZD/16/6) and the Scottish Funding Council (HR03006). The development of the GENOSCORES platform was supported by a Springboard Award (SBF006/1109) from the Academy of Medical Sciences, supported in turn by the Wellcome Trust, the UK Government Department of Business, Energy and Industrial Strategy, the British Heart Foundation, and Diabetes UK.

Declaration of interests

The authors declare no competing interests.

Received: December 22, 2022
Accepted: April 12, 2023
Published: May 9, 2023

Web resources

3D Genome Browser, http://3dgenome.fsm.northwestern.edu/view.php?method=Hi-C&species=human&assembly=hg38&source=inside&tissue=Liver_STL011&type=Leung_2015-raw&resolution=40&c_url=&transfer=&chr=chr19&start=44500000&end=48800000&sessionID=&browser=none

bedtools, https://bedtools.readthedocs.io/en/latest/index.html

duoHMM, https://mathgen.stats.ox.ac.uk/genetics_software/duohmm/duohmm.html

Eagle, https://alkesgroup.broadinstitute.org/Eagle/Eagle_manual.html

LiftOver, https://genome.sph.umich.edu/wiki/LiftOver

PLINK, https://www.cog-genomics.org/plink/

SNPTEST, https://www.well.ox.ac.uk/%7Egav/snptest/

References

1. Boyle, E.A., Li, Y.I., and Pritchard, J.K. (2017). An expanded view of complex traits: from polygenic to omnigenic. Cell *169*, 1177–1186. https://doi.org/10.1016/j.cell.2017.05.038.
2. Kirsten, H., Al-Hasani, H., Holdt, L., Gross, A., Beutner, F., Krohn, K., Horn, K., Ahnert, P., Burkhardt, R., Reiche, K., et al. (2015). Dissecting the genetics of the human transcriptome identifies novel trait-related trans-eQTLs and corroborates the regulatory relevance of non-protein coding loci. Hum. Mol. Genet. *24*, 4746–4763. https://doi.org/10.1093/hmg/ddv194.
3. Liu, X., Li, Y.I., and Pritchard, J.K. (2019). Trans Effects on Gene Expression Can Drive Omnigenic Inheritance. Cell *177*, 1022–1034.e6. https://doi.org/10.1016/j.cell.2019.04.014.
4. Võsa, U., Claringbould, A., Westra, H.-J., Bonder, M.J., Deelen, P., Zeng, B., Kirsten, H., Saha, A., Kreuzhuber, R., Yazar, S., et al. (2021). Large-scale cis- and trans-eQTL analyses identify thousands of genetic loci and polygenic scores that regulate blood gene expression. Nat. Genet. *53*, 1300–1310. https://doi.org/10.1038/s41588-021-00913-z.
5. Clayton, D.G. (2009). Prediction and interaction in complex disease genetics: Experience in type 1 diabetes. PLoS Genet. *5*, e1000540. https://doi.org/10.1371/journal.pgen.1000540.
6. Winkler, C., Krumsiek, J., Buettner, F., Angermüller, C., Giannopoulou, E.Z., Theis, F.J., Ziegler, A.-G., and Bonifacio, E. (2014). Feature ranking of type 1 diabetes susceptibility genes improves prediction of type 1 diabetes. Diabetologia *57*, 2521–2529. https://doi.org/10.1007/s00125-014-3362-1.
7. Akbar, T., McGurnaghan, S., Palmer, C.N.A., Livingstone, S.J., Petrie, J., Chalmers, J., Lindsay, R.S., McKnight, J.A., Pearson, D.W.M., Patrick, A.W., et al. (2017). Cohort profile: scottish diabetes research network type 1 bioresource study (SDRNT1BIO). Int. J. Epidemiol. *46*. 796-796i. https://doi.org/10.1093/ije/dyw152.
8. McKeigue, P.M., Spiliopoulou, A., McGurnaghan, S., Colombo, M., Blackbourn, L., McDonald, T.J., Onengut-Gomuscu, S., Rich, S.S., A Palmer, C.N., McKnight, J.A., et al. (2019). Persistent C-peptide secretion in Type 1 diabetes and its relationship to the genetic architecture of diabetes. BMC Med. *17*, 165. https://doi.org/10.1186/s12916-019-1392-8.
9. Smith, B.H., Campbell, H., Blackwood, D., Connell, J., Connor, M., Deary, I.J., Dominiczak, A.F., Fitzpatrick, B., Ford, I., Jackson, C., et al. (2006). Generation Scotland: The Scottish Family Health Study; a new resource for researching genes and heritability. BMC Med. Genet. *7*, 74. https://doi.org/10.1186/1471-2350-7-74.
10. Goldstein, J.I., Crenshaw, A., Carey, J., Grant, G.B., Maguire, J., Fromer, M., O'Dushlaine, C., Moran, J.L., Chambert, K., Stevens, C., et al. (2012). zCall: A rare variant caller for array-based genotyping. Bioinformatics *28*, 2543–2545. https://doi.org/10.1093/bioinformatics/bts479.
11. Clarke, L., Fairley, S., Zheng-Bradley, X., Streeter, I., Perry, E., Lowy, E., Tassé, A.M., and Flicek, P. (2017). The international Genome sample resource (IGSR): a worldwide collection of genome variation incorporating the 1000 Genomes Project data. Nucleic Acids Res. *45*, D854–D859. https://doi.org/10.1093/nar/gkw829.
12. UK10K Consortium, Walter, K., Min, J.L., Huang, J., Crooks, L., Memari, Y., McCarthy, S., Perry, J.R.B., Xu, C., Futema, M., et al. (2015). The UK10K project identifies rare variants in health and disease. Nature *526*, 82–90. https://doi.org/10.1038/nature14962.
13. Loh, P.-R., Palamara, P.F., and Price, A.L. (2016). Fast and accurate long-range phasing in a UK Biobank cohort. Nat. Genet. *48*, 811–816. https://doi.org/10.1038/ng.3571.
14. O'Connell, J., Gurdasani, D., Delaneau, O., Pirastu, N., Ulivi, S., Cocca, M., Traglia, M., Huang, J., Huffman, J.E., Rudan, I., et al. (2014). A general approach for haplotype phasing across the full spectrum of relatedness. PLoS Genet. *10*, e1004234. https://doi.org/10.1371/journal.pgen.1004234.
15. Marchini, J., Howie, B., Myers, S., McVean, G., and Donnelly, P. (2007). A new multipoint method for genome-wide association studies by imputation of genotypes. Nat. Genet. *39*, 906–913. https://doi.org/10.1038/ng2088.
16. Robertson, C.C., Inshaw, J.R.J., Onengut-Gumuscu, S., Chen, W.-M., Santa Cruz, D.F., Yang, H., Cutler, A.J., Crouch, D.J.M., Farber, E., Bridges, S.L., et al. (2021). Fine-mapping, trans-ancestral and genomic analyses identify causal variants, cells, genes and drug targets for type 1 diabetes. Nat. Genet. *53*, 962–971. https://doi.org/10.1038/s41588-021-00880-5.
17. Vujkovic, M., Keaton, J.M., Lynch, J.A., Miller, D.R., Zhou, J., Tcheandjieu, C., Huffman, J.E., Assimes, T.L., Lorenz, K., Zhu, X., et al. (2020). Discovery of 318 new risk loci for type 2 diabetes and related vascular outcomes among 1.4 million participants in a multi-ancestry meta-analysis. Nat. Genet. *52*, 680–691. https://doi.org/10.1038/s41588-020-0637-y.
18. Howe, K.L., Achuthan, P., Allen, J., Allen, J., Alvarez-Jarreta, J., Amode, M.R., Armean, I.M., Azov, A.G., Bennett, R., Bhai, J.,

et al. (2021). Ensembl 2021. Nucleic Acids Res. *49*, D884–D891. https://doi.org/10.1093/nar/gkaa942.

19. Spiliopoulou, A., Colombo, M., Plant, D., Nair, N., Cui, J., Coenen, M.J., Ikari, K., Yamanaka, H., Saevarsdottir, S., Padyukov, L., et al. (2019). Association of response to TNF inhibitors in rheumatoid arthritis with quantitative trait loci for CD40 and CD39. Ann. Rheum. Dis. *78*, 1055–1061. https://doi.org/10.1136/annrheumdis-2018-214877.
20. Fehrmann, R.S.N., Jansen, R.C., Veldink, J.H., Westra, H.-J., Arends, D., Bonder, M.J., Fu, J., Deelen, P., Groen, H.J.M., Smolonska, A., et al. (2011). Trans-eQTLs reveal that independent genetic variants associated with a complex phenotype converge on intermediate genes, with a major role for the HLA. PLoS Genet. *7*, e1002197. https://doi.org/10.1371/journal.pgen.1002197.
21. McKeigue, P. (2019). Quantifying performance of a diagnostic test as the expected information for discrimination: Relation to the C-statistic. Stat. Methods Med. Res. *28*, 1841–1851. https://doi.org/10.1177/0962280218776989.
22. Hill, M.O. (1973). Diversity and evenness: a unifying notation and its consequences. Ecology *54*, 427–432. https://doi.org/10.2307/1934352.
23. Ahola-Olli, A.V., Würtz, P., Havulinna, A.S., Aalto, K., Pitkänen, N., Lehtimäki, T., Kähönen, M., Lyytikäinen, L.P., Raitoharju, E., Seppälä, I., et al. (2017). Genome-wide association study identifies 27 loci influencing concentrations of circulating cytokines and growth factors. Am. J. Hum. Genet. *100*, 40–50. https://doi.org/10.1016/j.ajhg.2016.11.007.
24. Sun, B.B., Maranville, J.C., Peters, J.E., Stacey, D., Staley, J.R., Blackshaw, J., Burgess, S., Jiang, T., Paige, E., Surendran, P., et al. (2018). Genomic atlas of the human plasma proteome. Nature *558*, 73–79. https://doi.org/10.1038/s41586-018-0175-2.
25. Ferkingstad, E., Sulem, P., Atlason, B.A., Sveinbjornsson, G., Magnusson, M.I., Styrmisdottir, E.L., Gunnarsdottir, K., Helgason, A., Oddsson, A., Halldorsson, B.V., et al. (2021). Large-scale integration of the plasma proteome with genetics and disease. Nat. Genet. *53*, 1712–1721. https://doi.org/10.1038/s41588-021-00978-w.
26. Folkersen, L., Fauman, E., Sabater-Lleal, M., Strawbridge, R.J., Frånberg, M., Sennblad, B., Baldassarre, D., Veglia, F., Humphries, S.E., Rauramaa, R., et al. (2017). Mapping of 79 loci for 83 plasma protein biomarkers in cardiovascular disease. PLoS Genet. *13*, e1006706. https://doi.org/10.1371/journal.pgen.1006706.
27. Hillary, R.F., Trejo-Banos, D., Kousathanas, A., McCartney, D.L., Harris, S.E., Stevenson, A.J., Patxot, M., Ojavee, S.E., Zhang, Q., Liewald, D.C., et al. (2020). Multi-method genome- and epigenome-wide studies of inflammatory protein levels in healthy older adults. Genome Med. *12*, 60. https://doi.org/10.1186/s13073-020-00754-1.
28. Oram, R.A., Patel, K., Hill, A., Shields, B., McDonald, T.J., Jones, A., Hattersley, A.T., and Weedon, M.N. (2016). A type 1 diabetes genetic risk score can aid discrimination between type 1 and type 2 diabetes in young adults. Diabetes Care *39*, 337–344. https://doi.org/10.2337/dc15-1111.
29. Orrù, V., Steri, M., Sole, G., Sidore, C., Virdis, F., Dei, M., Lai, S., Zoledziewska, M., Busonero, F., Mulas, A., et al. (2013). Genetic variants regulating immune cell levels in health and disease. Cell *155*, 242–256. https://doi.org/10.1016/j.cell.2013.08.041.
30. Roederer, M., Quaye, L., Mangino, M., Beddall, M.H., Mahnke, Y., Chattopadhyay, P., Tosi, I., Napolitano, L., Terranova Barberio, M., Menni, C., et al. (2015). The Genetic Architecture of the Human Immune System: A Bioresource for Autoimmunity and Disease Pathogenesis. Cell *161*, 387–403. https://doi.org/10.1016/j.cell.2015.02.046.
31. Todd, J.A., Walker, N.M., Cooper, J.D., Smyth, D.J., Downes, K., Plagnol, V., Bailey, R., Nejentsev, S., Field, S.F., Payne, F., et al. (2007). Robust associations of four new chromosome regions from genome-wide analyses of type 1 diabetes. Nat. Genet. *39*, 857–864. https://doi.org/10.1038/ng2068.
32. Hakonarson, H., Qu, H.-Q., Bradfield, J.P., Marchand, L., Kim, C.E., Glessner, J.T., Grabs, R., Casalunovo, T., Taback, S.P., Frackelton, E.C., et al. (2008). A novel susceptibility locus for type 1 diabetes on Chr12q13 identified by a genome-wide association study. Diabetes *57*, 1143–1146. https://doi.org/10.2337/db07-1305.
33. Wang, Y., Song, F., Zhang, B., Zhang, L., Xu, J., Kuang, D., Li, D., Choudhary, M.N.K., Li, Y., Hu, M., et al. (2018). The 3D genome browser: a web-based browser for visualizing 3D genome organization and long-range chromatin interactions. Genome Biol. *19*, 151. https://doi.org/10.1186/s13059-018-1519-9.
34. Johnson, M.B., Hattersley, A.T., and Flanagan, S.E. (2016). Monogenic autoimmune diseases of the endocrine system. Lancet Diabetes Endocrinol. *4*, 862–872. https://doi.org/10.1016/S2213-8587(16)30095-X.
35. Ueda, H., Howson, J.M.M., Esposito, L., Heward, J., Snook, H., Chamberlain, G., Rainbow, D.B., Hunter, K.M.D., Smith, A.N., Di Genova, G., et al. (2003). Association of the T-cell regulatory gene CTLA4 with susceptibility to autoimmune disease. Nature *423*, 506–511. https://doi.org/10.1038/nature01621.
36. Gerold, K.D., Zheng, P., Rainbow, D.B., Zernecke, A., Wicker, L.S., and Kissler, S. (2011). The soluble CTLA-4 splice variant protects from type 1 diabetes and potentiates regulatory T-cell function. Diabetes *60*, 1955–1963. https://doi.org/10.2337/db11-0130.
37. Westra, H.-J., Peters, M.J., Esko, T., Yaghootkar, H., Schurmann, C., Kettunen, J., Christiansen, M.W., Fairfax, B.P., Schramm, K., Powell, J.E., et al. (2013). Systematic identification of trans-eQTLs as putative drivers of known disease associations. Nat. Genet. *45*, 1238–1243. https://doi.org/10.1038/ng.2756.
38. Fontenot, J.D., Gavin, M.A., and Rudensky, A.Y. (2003). Foxp3 programs the development and function of CD4+CD25+ regulatory T cells. Nat. Immunol. *4*, 330–336. https://doi.org/10.1038/ni904.
39. Chikuma, S., and Bluestone, J.A. (2007). Expression of CTLA-4 and FOXP3 in cis protects from lethal lymphoproliferative disease. Eur. J. Immunol. *37*, 1285–1289. https://doi.org/10.1002/eji.200737159.
40. Chang, J.-H., Kim, Y.-J., Han, S.-H., and Kang, C.-Y. (2009). IFN-gamma-STAT1 signal regulates the differentiation of inducible Treg: potential role for ROS-mediated apoptosis. Eur. J. Immunol. *39*, 1241–1251. https://doi.org/10.1002/eji.200838913.
41. Henderson, J.G., Opejin, A., Jones, A., Gross, C., and Hawiger, D. (2015). CD5 instructs extrathymic regulatory T cell development in response to self and tolerizing antigens. Immunity *42*, 471–483. https://doi.org/10.1016/j.immuni.2015.02.010.
42. Chaudhry, A., Samstein, R.M., Treuting, P., Liang, Y., Pils, M.C., Heinrich, J.-M., Jack, R.S., Wunderlich, F.T., Brüning, J.C., Müller, W., and Rudensky, A.Y. (2011). Interleukin-10 signaling in regulatory T cells is required for suppression of Th17 cell-mediated inflammation. Immunity *34*, 566–578. https://doi.org/10.1016/j.immuni.2011.03.018.

43. Alvisi, G., Termanini, A., Soldani, C., Portale, F., Carriero, R., Pilipow, K., Costa, G., Polidoro, M., Franceschini, B., Malenica, I., et al. (2022). Multimodal single-cell profiling of intrahepatic cholangiocarcinoma defines hyperactivated Tregs as a potential therapeutic target. J. Hepatol. *77*, 1359–1372. https://doi.org/10.1016/j.jhep.2022.05.043.
44. Lundholm, M., Mayans, S., Motta, V., Löfgren-Burström, A., Danska, J., and Holmberg, D. (2010). Variation in the Cd3 zeta (Cd247) gene correlates with altered T cell activation and is associated with autoimmune diabetes. J. Immunol. *184*, 5537–5544. https://doi.org/10.4049/jimmunol.0904012.
45. Hong, C.-S., Park, M.-R., Sun, E.-G., Choi, W., Hwang, J.-E., Bae, W.-K., Rhee, J.H., Cho, S.-H., and Chung, I.-J. (2019). Gal-3BP Negatively Regulates NF-κB Signaling by Inhibiting the Activation of TAK1. Front. Immunol. *10*, 1760.
46. Van Kaer, L., Wu, L., and Joyce, S. (2016). Mechanisms and Consequences of Antigen Presentation by CD1. Trends Immunol. *37*, 738–754. https://doi.org/10.1016/j.it.2016.08.011.
47. Phillips, R. (2022). FOXP3 splice variant is associated with autoimmune disease. Nat. Rev. Rheumatol. *18*, 493. https://doi.org/10.1038/s41584-022-00818-z.
48. Cotsapas, C., Voight, B.F., Rossin, E., Lage, K., Neale, B.M., Wallace, C., Abecasis, G.R., Barrett, J.C., Behrens, T., Cho, J., et al. (2011). Pervasive Sharing of Genetic Effects in Autoimmune Disease. PLoS Genet. *7*, e1002254. https://doi.org/10.1371/journal.pgen.1002254.
49. Gur, C., Porgador, A., Elboim, M., Gazit, R., Mizrahi, S., Stern-Ginossar, N., Achdout, H., Ghadially, H., Dor, Y., Nir, T., et al. (2010). The activating receptor NKp46 is essential for the development of type 1 diabetes. Nat. Immunol. *11*, 121–128. https://doi.org/10.1038/ni.1834.
50. Takeuchi, A., Itoh, Y., Takumi, A., Ishihara, C., Arase, N., Yokosuka, T., Koseki, H., Yamasaki, S., Takai, Y., Miyoshi, J., et al. (2009). CRTAM confers late-stage activation of CD8+ T cells to regulate retention within lymph node. J. Immunol. *183*, 4220–4228. https://doi.org/10.4049/jimmunol.0901248.
51. Bettini, M., Szymczak-Workman, A.L., Forbes, K., Castellaw, A.H., Selby, M., Pan, X., Drake, C.G., Korman, A.J., and Vignali, D.A.A. (2011). Accelerated autoimmune diabetes in the absence of LAG-3. J. Immunol. *187*, 3493–3498. https://doi.org/10.4049/jimmunol.1100714.
52. Burke, S.J., Karlstad, M.D., Eder, A.E., Regal, K.M., Lu, D., Burk, D.H., and Collier, J.J. (2016). Pancreatic β-Cell production of CXCR3 ligands precedes diabetes onset. Biofactors *42*, 703–715. https://doi.org/10.1002/biof.1304.
53. Yamada, Y., Okubo, Y., Shimada, A., Oikawa, Y., Yamada, S., Narumi, S., Matsushima, K., and Itoh, H. (2012). Acceleration of diabetes development in CXC chemokine receptor 3 (CXCR3)-deficient NOD mice. Diabetologia *55*, 2238–2245. https://doi.org/10.1007/s00125-012-2547-8.
54. Kotarsky, K., Sitnik, K.M., Stenstad, H., Kotarsky, H., Schmidtchen, A., Koslowski, M., Wehkamp, J., and Agace, W.W. (2010). A novel role for constitutively expressed epithelial-derived chemokines as antibacterial peptides in the intestinal mucosa. Mucosal Immunol. *3*, 40–48. https://doi.org/10.1038/mi.2009.115.
55. Shan, Z., Xu, B., Mikulowska-Mennis, A., and Michie, S.A. (2014). CCR7 directs the recruitment of T cells into inflamed pancreatic islets of nonobese diabetic (NOD) mice. Immunol. Res. *58*, 351–357. https://doi.org/10.1007/s12026-014-8500-9.
56. Martínez-Vicente, P., Farré, D., Sánchez, C., Alcamí, A., Engel, P., and Angulo, A. (2019). Subversion of natural killer cell responses by a cytomegalovirus-encoded soluble CD48 decoy receptor. PLoS Pathog. *15*, e1007658. https://doi.org/10.1371/journal.ppat.1007658.
57. Martinez, V.G., Escoda-Ferran, C., Tadeu Simões, I., Arai, S., Orta Mascaró, M., Carreras, E., Martínez-Florensa, M., Yelamos, J., Miyazaki, T., and Lozano, F. (2014). The macrophage soluble receptor AIM/Api6/CD5L displays a broad pathogen recognition spectrum and is involved in early response to microbial aggression. Cell. Mol. Immunol. *11*, 343–354. https://doi.org/10.1038/cmi.2014.12.
58. Yip, L., Creusot, R.J., Pager, C.T., Sarnow, P., and Fathman, C.G. (2013). Reduced DEAF1 function during type 1 diabetes inhibits translation in lymph node stromal cells by suppressing Eif4g3. J. Mol. Cell Biol. *5*, 99–110. https://doi.org/10.1093/jmcb/mjs052.
59. Mensah-Brown, E.P.K., Shahin, A., Al-Shamisi, M., Wei, X., and Lukic, M.L. (2006). IL-23 leads to diabetes induction after subdiabetogenic treatment with multiple low doses of streptozotocin. Eur. J. Immunol. *36*, 216–223. https://doi.org/10.1002/eji.200535325.
60. Shapiro, M.R., Yeh, W.-I., Longfield, J.R., Gallagher, J., Infante, C.M., Wellford, S., Posgai, A.L., Atkinson, M.A., Campbell-Thompson, M., Lieberman, S.M., et al. (2020). CD226 Deletion Reduces Type 1 Diabetes in the NOD Mouse by Impairing Thymocyte Development and Peripheral T Cell Activation. Front. Immunol. *11*, 2180. https://doi.org/10.3389/fimmu.2020.02180.
61. Dutta, D., He, Y., Saha, A., Arvanitis, M., Battle, A., and Chatterjee, N. (2022). Aggregative trans-eQTL analysis detects trait-specific target gene sets in whole blood. Nat. Commun. *13*, 4323. https://doi.org/10.1038/s41467-022-31845-9.
62. Mathieson, I. (2021). The omnigenic model and polygenic prediction of complex traits. Am. J. Hum. Genet. *108*, 1558–1563. https://doi.org/10.1016/j.ajhg.2021.07.003.

The American Journal of Human Genetics, Volume *110*

Supplemental information

Genome-wide aggregated *trans*-effects on risk of type 1 diabetes: A test of the "omnigenic" sparse effector hypothesis of complex trait genetics

Andrii Iakovliev, Stuart J. McGurnaghan, Caroline Hayward, Marco Colombo, Debby Lipschutz, Athina Spiliopoulou, Helen M. Colhoun, and Paul M. McKeigue

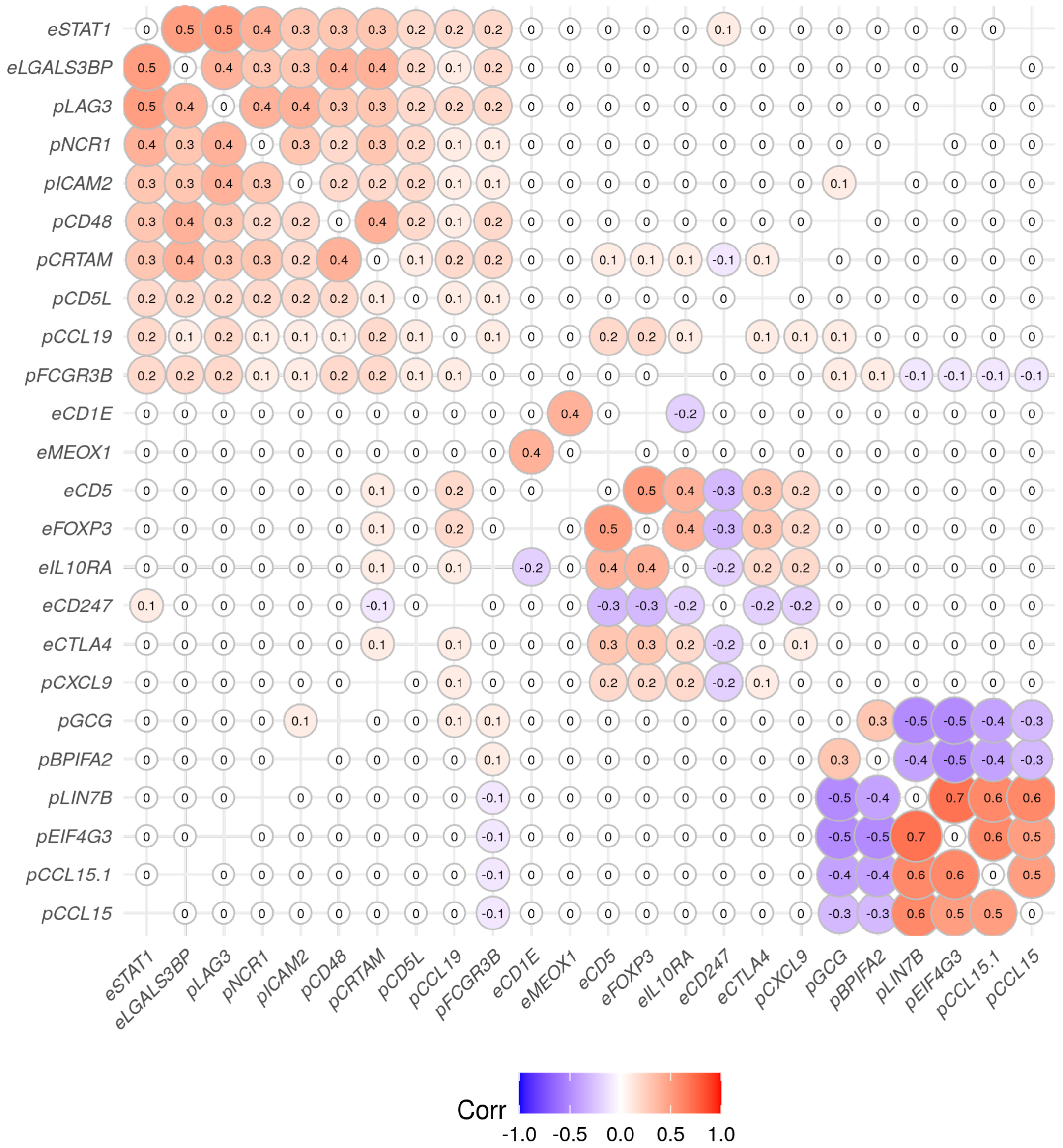

Fig S1. Heat map of correlations (in control group) between *trans*- scores for putative effector genes

The correlations between eQTL scores for genes identified in Table 2 and pQTL scores for genes identified in Table 4 is reported. Rows and columns are ordered by hierarchical clustering. The prefixes *e* and *p* denote scores for gene expression scores and circulating protein levels respectively.

Table S1. *cis*- eQTL or pQTLassociations with T1D detected at $p < 0.01$ in the 12q13 and 12q24 regions.

Gene	Start position (Mb)	*cis*-eQTL		*cis*-pQTL	
		Log odds ratio	p-value	Log odds ratio	p-value
**12q13 region**					
RAB5B	55.97	0.14	9×10^{-14}	.	.
ERBB3	56.08	.	.	-0.14	6×10^{-13}
ERBB3	56.08	.	.	-0.13	3×10^{-11}
IL23A	56.33	-0.16	2×10^{-17}	.	.
GLS2	56.47	-0.05	0.006	.	.
**12q24 region**					
ISCU	108.56	-0.01	0.4	0.14	1×10^{-12}
SELPLG	108.62	0.02	0.3	0.11	2×10^{-9}
GLTP	109.85	.	.	0.11	6×10^{-9}
PPTC7	110.53	-0.14	4×10^{-13}	.	.
PHETA1	111.36	-0.06	0.002	.	.
SH2B3	111.41	0.08	2×10^{-5}	.	.
ALDH2	111.77	0.01	0.5	0.10	1×10^{-7}
TRAFD1	112.13	0.11	4×10^{-8}	.	.
OAS3	112.94	-0.05	0.005	.	.
OAS2	112.98	-0.06	6×10^{-4}	.	.
SDSL	113.42	0.11	2×10^{-9}	0.03	0.2
TBX5	114.35	.	.	0.12	1×10^{-9}

Table S4. Associations of T1D with genome-wide scores for regulatory T cell phenotypes computed using weights from SardiNIA study[1].

	Absolute count		% of parent lineage		% of grandparent lineage	
Cell phenotype	Log odds ratio	p-value	Log odds ratio	p-value	Log odds ratio	p-value
CD39+ (resting & activated) CD4+ Treg	.	.	-0.03	0.08	-0.02	0.2
CD39+ (secreting & activated) CD4+ Treg	-0.02	0.2	-0.01	0.3	-0.01	0.4
CD39+ CD4+ Treg	-0.01	0.7	-0.01	0.4	0.00	0.8
CD39+ activated CD4+ Treg	-0.03	0.07	-0.01	0.3	-0.02	0.3
CD39+ resting CD4+ Treg	-0.02	0.1	-0.03	0.02	-0.01	0.4
CD39+ secreting CD4+ Treg	-0.02	0.2	-0.02	0.2	0.00	0.9
CD4+ Treg	-0.08	0.03	-0.06	0.03	-0.01	0.8
activated CD4+ Treg	.	.	-0.05	0.3	-0.01	0.7
resting & activated CD4+ Treg	0.03	0.3	0.02	0.3	0.00	0.9
resting CD4+ Treg	0.02	0.6	-0.01	0.7	0.02	0.6
secreting & activated CD4+ Treg	-0.07	0.008	0.00	0.8	-0.05	0.04
secreting CD4+ Treg	-0.05	0.08	-0.01	0.6	-0.02	0.4

Immune cell phenotypes are defined in Orrù et al. (2013) supplementary information[1].

Table S6. *Trans*-pQTLs near T1D-associated genes contributing to *trans*- scores in Table 4.

Chr	Start position (Mb)	End position (Mb)	Genes to which T1D association with SNPs within 200 kb of the *trans*-pQTL region was attributed	Genes encoding proteins with T1D-associated *trans*- scores
1	113.53	113.99	*PTPN22, MAGI3, PHTF1, RSBN1*	*CCL19, CRTAM, CXCL9*
1	206.87	206.97	*IL10*	*BPIFA2*
2	60.62	60.62	*BCL11A, KIAA1841*	*CXCL9*
2	191.09	191.14	*STAT4*	*CRTAM*
2	203.88	203.92	*CTLA4*	*CCL19*
3	45.97	47.54	*CCR9*	*CXCL9, CRTAM, CD48*
4	122.71	122.71	*KIAA1109*	*CXCL9*
5	86.75	86.79	*AC008394.1*	*CCL15*
6	35.42	35.42	*TULP1*	*CXCL9*
7	20.27	20.27	*ITGB8*	*GCG*
7	50.23	50.41	*IKZF1, FIGNL1*	*FCGR3B, LAG3, CD5L*
8	126.91	127.22	*PVT1*	*CD5L*
9	133.17	133.73	*CEL, RALGDS, OBP2B*	*ICAM2, CCL15, BPIFA2, GCG*
10	88.05	88.05	*RNLS*	*ICAM2*
11	2.51	2.53	*INS, TH*	*CXCL9*
11	128.34	128.54	*FLI1*	*CRTAM*
12	9.07	9.07	*KLRG1, PHC1*	*CD48*
12	110.82	112.88	*ATXN2, BRAP*	*CD5L, NCR1, CD48, CXCL9, LAG3, FCGR3B, CRTAM, ICAM2, CCL19*
12	112.70	112.75	*ATXN2*	*LAG3*
13	98.79	98.79	*GPR183*	*CXCL9*
14	100.70	100.72	*AL117190.2*	*NCR1*
16	30.16	31.14	*MAPK3*	*CD5L*
17	6.83	7.64	*ACAP1, DNAH2*	*CD48, FCGR3B, LAG3, CRTAM, CD5L*
17	39.24	39.97	*GSDMB*	*FCGR3B*
17	45.11	46.79	*STH, NSF*	*CCL15, CD48, CD5L, CRTAM*
17	47.28	48.25	*PRR15L, SKAP1*	*FCGR3B, GCG*
19	46.35	46.80	*PRDK2*	*NCR1, FCGR3B*
19	48.10	49.12	*FUT2*	*CCL15, BPIFA2, GCG, FCGR3B*
21	42.17	42.19	*UBASH3A*	*CXCL9*
21	42.88	42.88	*CFAP410*	*GCG*
22	29.73	30.20	*LIF*	*CCL19*

Supplementary references

1. Orrù, V., Steri, M., Sole, G., Sidore, C., Virdis, F., Dei, M., Lai, S., Zoledziewska, M., Busonero, F., Mulas, A., et al. (2013). Genetic Variants Regulating Immune Cell Levels in Health and Disease. Cell *155*, 242–256. 10.1016/j.cell.2013.08.041.

2. Roederer, M., Quaye, L., Mangino, M., Beddall, M.H., Mahnke, Y., Chattopadhyay, P., Tosi, I., Napolitano, L., Terranova Barberio, M., Menni, C., et al. (2015). The Genetic Architecture of the Human Immune System: A Bioresource for Autoimmunity and Disease Pathogenesis. Cell *161*, 387–403. 10.1016/j.cell.2015.02.046.
